# Identification and validation of iron metabolism genes in osteoporosis

**DOI:** 10.1186/s12920-023-01779-2

**Published:** 2024-01-02

**Authors:** Zutao Li, Jiangbo Xu, Shouyin Shi, Youlin Weng, Bin Guo, Lixin Che, Jungang Sun

**Affiliations:** Department of Orthopedic Trauma, People’s Hospital of Xinjiang Uygur Autonomous Region, Urumqi, 830001 Xinjiang China

**Keywords:** Osteoporosis, Iron metabolism, DEGs, Hub gene, Bioinformatics

## Abstract

**Background:**

Osteoporosis is the most common metabolic bone disease in humans. Exploring the expression difference of iron metabolism-related genes in osteoporosis can provide a new target for diagnosis and treatment.

**Methods:**

First, we used online databases to identify differentially expressed genes (DEGs) related to iron metabolism in patients with osteoporosis. The differential genes were comprehensively analyzed by bioinformatics method (GO, KEGG, GSEA, immune infiltration analysis, PPI). The expression levels of hub genes and important signaling pathways were verified by qRT-PCR and Western blotting.

**Results:**

A total of 23 iron metabolism-related genes with significant differences were identified, which were enriched in “regulation of protein dephosphorylation” and “negative regulation of protein dephosphorylation”. The GSEA results, heme metabolism and Myc targets v1 were among the top two pathways, both upregulated. The immune infiltration analysis revealed that the expressions of genes such as ABCA5, D2HGDH, GNAI2, and CTSW were correlated with the infiltration degree of significantly different cells. The PPI network contained 12 differentially expressed iron metabolism-related genes. Additionally, YWHAE, TGFB1, PPP1R15A, TOP2A, and CALR were mined as hub genes using the Cytoscape software. qRT PCR showed that the expression of TGF-β1, YWHAE, TOP2A and CALR increased. We also verified the expression of related proteins and genes in the oxidative stress signaling pathway by qRT PCR and Western blotting. The results showed that Mob1, YAP and TAZ molecules were highly expressed at the gene and protein levels.

**Conclusions:**

These differentially expressed iron metabolism-related genes could provide new potential targets for the diagnosis and treatment of osteoporosis.

**Supplementary Information:**

The online version contains supplementary material available at 10.1186/s12920-023-01779-2.

## Background

Osteoporosis is a chronic and systemic metabolic disease induced by multiple causes and mainly characterized by osteopenia, microstructural changes, and fractures [1]. With the increase in life expectancy across the globe, its incidence is expected to increase further. According to recent data, 3 million people over 50 suffer from osteoporotic fractures of varying degrees in the United States, and nearly 25 billion USD have been spent for treating this condition [2]. The pathogenesis of osteoporosis is still not fully understood.

Previous studies on osteoporosis mainly focused on the biological behaviors of vitamin D and calcium-phosphorus metabolism. However, an increasing number of studies have also suggested that abnormal iron metabolism is closely associated with the occurrence and development of osteoporosis [3, 4]. A study found that iron overload or deficiency can affect the proliferation and differentiation of osteoblasts and osteoclasts, resulting in decreased bone mass and increased risk of osteoporosis and fracture [5]. In addition, the incidence of osteoporosis caused by abnormal iron metabolism has been associated with the antioxidant/prooxidant equilibrium of cells [6]. However, little research has been conducted on iron metabolism and osteoporosis from a genetic and bioinformatics perspective.

In this study, we used the bioinformatics method to analyze the relevant data on osteoporosis and iron metabolism in existing databases, aiming to explore and identify differentially expressed iron metabolism-related genes in osteoporosis patients and carry out biological function analysis and other multi-dimensional analyses on the identified DEGs, as well as profoundly study the impacts of abnormal iron metabolism on osteoporosis at the genetic level.

## Methods

### Microarray data

**Fig. 1:**
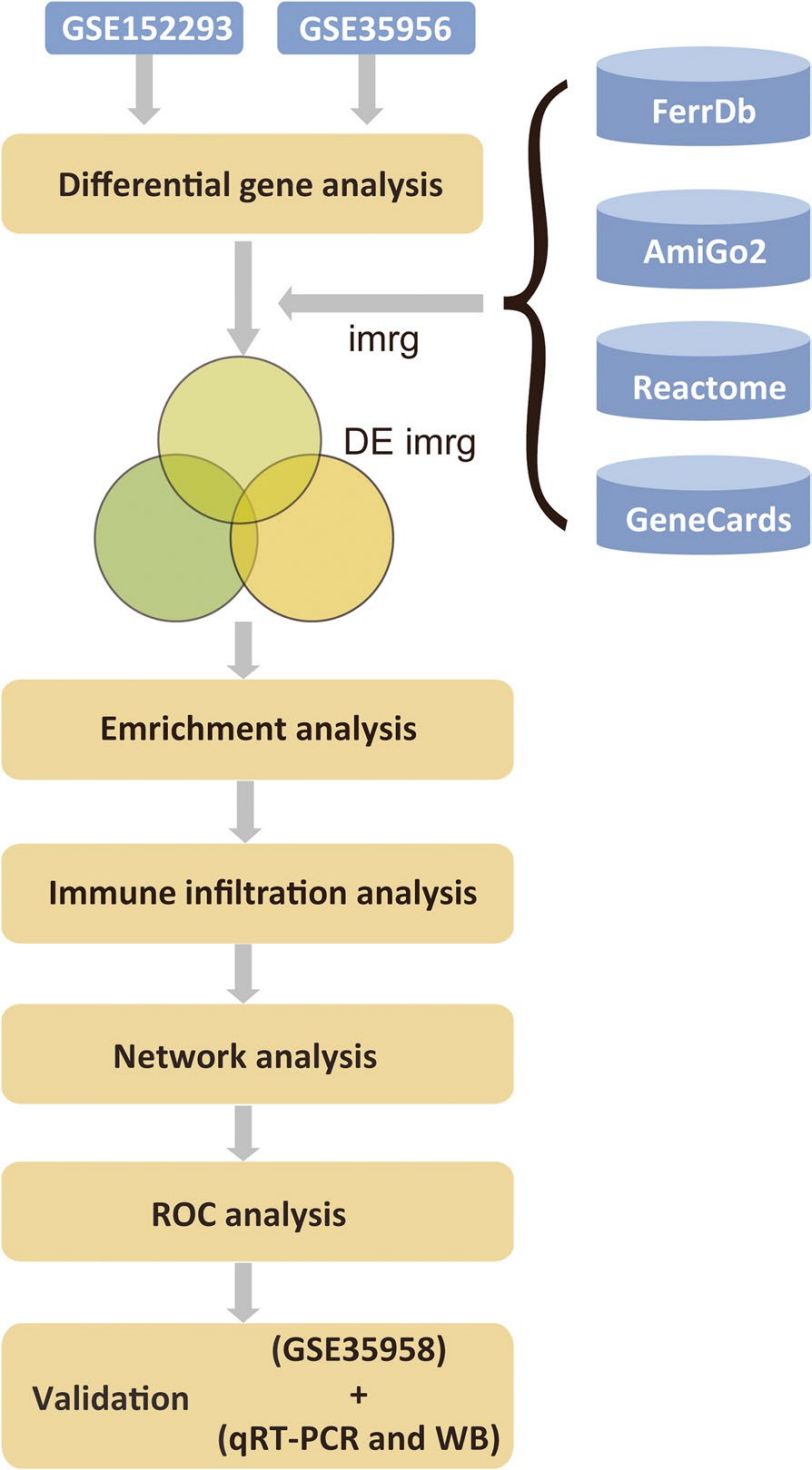
Study Workflow. IMRG: Iron metabolism-related genes, DE IMRG: Differentially expressed iron metabolism-related genes. qRT-PCR: quantitative reverse transcription PCR, WB: western blot

Data on osteoporosis with the following data numbers were downloaded: GSE152293 [7], GSE35956 [8] and GSE35958 [8] and the clinical information of corresponding samples was obtained from the Gene Expression Omnibus (GEO) database (https://www.ncbi.nlm.nih.gov/geo/) [9]; all samples were from Homo sapiens. The sequencing platforms were GPL11154, GPL570, and GPL570. GSE152293 included six patients, three with osteoporosis and three healthy patients; the GSE35956 included ten patients, five patients with osteoporosis and five healthy patients; the GSE35958 included nine patients, five patients with osteoporosis and four healthy patients. The data in GSE152293 and GSE35956 were used for analysis, and GSE35958 was used as a validation dataset to validate the obtained results (Fig. 1).

The studies involving human participants were reviewed and approved by Ethics Committee of People’s Hospital of Xin jiang Uygur Autonomous Region, (the license number: KY2022072245). The patients/participants provided their written informed consent to participate in this study.

Next, the R package GEOquery (v2.60.0) [10] was used to download expression data and the limma package (v3.48.3) [11] was used for between-sample normalization processing, with box diagrams for visualization. The R package FactoMineR (v2.4) [12] was used for Principal Component Analysis (PCA) and visualization.

Iron metabolism-related genes were from the ferroptosis phenotype database FerrDb (http://www.zhounan.org/ferrdb) [13], “iron absorption and transport” (R-HSA‐917,937) from the Reactome database (https://reactome.org/) [14], “intracellular iron homeostasis” (GO:0006879) from the AmiGo2 database (http://amigo.geneontology.org/amigo/landing) and “Iron metabolism” as iron metabolism-related genes (IRG) from GeneCards database (https://www.genecards.org) [15].

### Identification of metabolism-related genes

To reveal the differences in gene expressions between patients with osteoporosis and healthy patients, we analyzed the differential expressions of genes between groups with the R package limma [11]; |log2 fold change| (|log2FC|) ≥ 1 and *P*-value < 0.05 were set as the threshold for DEGs. Genes with log2FC > 1 and *P*-value < 0.05 were DEGs with upregulated expressions and genes with log2FC < -1 and *P*-value < 0.05 represented DEGs with downregulated expressions. The intersection of the upregulated genes in GSE152293 and GSE35956 and iron metabolism-related genes represented upgraded metabolism-related genes, and the intersection of the downregulated genes in GSE152293 and GSE35956 and iron metabolism-related genes represented downregulated metabolism-related genes. We used the R package ggplot2 (v3.3.5) [16] to create volcano plots and the P package pheatmap (v1.0.12) [17] to create heat maps for visualization.

### Gene enrichment analysis

Gene Ontology (GO) analyzes the biological process (BP), molecular function (MF), and cellular component (CC) [18]. Kyoto Encyclopedia of Genes and Genomes (KEGG) is a widely used database that stores information on genomes, biological pathways, diseases, and drugs [19]. GO function annotation and KEGG biological pathway enrichment analysis were performed using the R package clusterProfiler (v4.0.5) [20] to identify significantly enriched biological processes and pathways. The enrichment results were visualized through the R package GOplot (v1.0.2) [21], and the significance thresholds for enrichment analysis were all set as *P* < 0.05.

Gene Set Enrichment Analysis (GSEA) is a calculation method used to determine whether a pre-defined set of genes shows statistically significant differences between two biological states. It is typically used for estimating changes in pathways and biological process activity in expression dataset samples [22]. To study the differences in biological processes between the two groups of patients, we downloaded reference gene sets from the MSigDB database [23] (https://www.gsea-msigdb.org/gsea/msigdb/) based on gene expression profile datasets. Then, the GSEA method in the R package clusterProfiler (v4.0.5) [20] was used for enrichment analysis and visualization of these datasets. The mean logFC value was taken as the logFC value, and a *P*-value < 0.05 indicated statistical significance.

### Immune infiltration analysis

To further explore the differences in the immune infiltration degree between osteoporosis patients and the healthy group, CIBERSORT software [24] was used to assess immune cells’ infiltration degree. The content of 22 types of immune cells in each patient was calculated based on the LM22 background gene set provided by the CIBERSORT website (https://cibersort.stanford.edu/), reflecting the infiltration level. The results were displayed as box plots and stacked bar charts, the latter being created with the R package ggplot2 (v3.3.5) [16]. Additionally, scatter diagrams between significantly different immune cell infiltration levels with *P*-value < 0.05 and differentially expressed iron metabolism expression values were created using the R package ggExtra (v0.9) [25], and correlation curves were also fitted.

### Network construction and analysis

A protein-protein interaction (PPI) network was constructed using the STRING database [26] (https://www.string-db.org), with the above genes as input and the default value of 0.4 as the confidence threshold. Next, the PPI network was exported and further analyzed through the Cytoscape software (v3.8.2) [27]. The network properties of each node were calculated, hub nodes were mined using the MCC algorithm of the cytoHubba plug-in (v0.1) [28], the nodes were scored and sorted in descending order according to the MCC results, and the top five nodes were selected as hub nodes. Then, based on the miRNet database [29](https://www.mirnet.ca), the five hub nodes were further predicted at the miRNA and transcription factor levels. The target information of small-molecule drugs was downloaded from DrugBank [30] (https://go.drugbank.com/drugs), and small-molecule drugs bound to these hub nodes were predicted accordingly. After exporting the prediction results, Cytoscape (v3.8.2) [31] was used for processing and drawing diagrams.

### Validation

GSE35958 was used as a dataset to validate the above results. Differential expression analysis was performed using the same steps adopted for the datasets of GSE152293 and GSE25958. Then, GSE152293 and GSE25958 were intersected to identify differentially expressed iron metabolism-related genes, followed by GO and KEGG enrichment analysis. Next, the logFC value of GSE35958 was averaged with the logFC values of GSE152293 and GSE152293 for GSEA analysis. Finally, immune infiltration and network analysis were performed.

### ROC analysis

To further analyze the differences in differentially expressed iron metabolism-related genes between patients with osteoporosis and the healthy group, we used the pROC package (v1.18.0) [29] to calculate the ROC curves for these genes and groups and the area under the curve (AUC). Next, validation was performed on the validation dataset GSE35958 to further confirm the robustness of model prediction. Meanwhile, ROC curves were created, and the AUC was calculated to assess the performance of the models.

### qRT-PCR and western blotting

In order to verify the results of bioinformatics analysis, we collected bone tissue samples from 3 patients with osteoporosis (OP group) and 3 normal contrast patients (NC group) who came to our hospital for treatments. The bone tissue samples were all derived from the femoral head tissues of patients with femoral neck fractures treated in our hospital who received a total hip replacement in our hospital. All the subjects underwent scanning of the total lumbar spine (L1–L4), total hip, and femoral neck by dual X-ray absorptiometry (DXA). According to the WHO diagnostic classification, osteoporosis was defined by BMD at the hip or lumbar spine T-score≤ -2.5 SD. The BMD diagnosis of normal was based on the T-score of the lumbar spine or hip at -1.0 SD and above. The qRT-PCR and Western blotting methods were used to verify the hub genes analyzed using bioinformatics.

### Statistical analysis

All data calculation and statistical analysis were performed in the R language (v4.1.0). The group differences between independent variables were compared using a t-test. Two-sided *P* < 0.05 was considered statistically significant.

## Results

### Differentially expressed genes (DEGs)

**Fig. 2:**
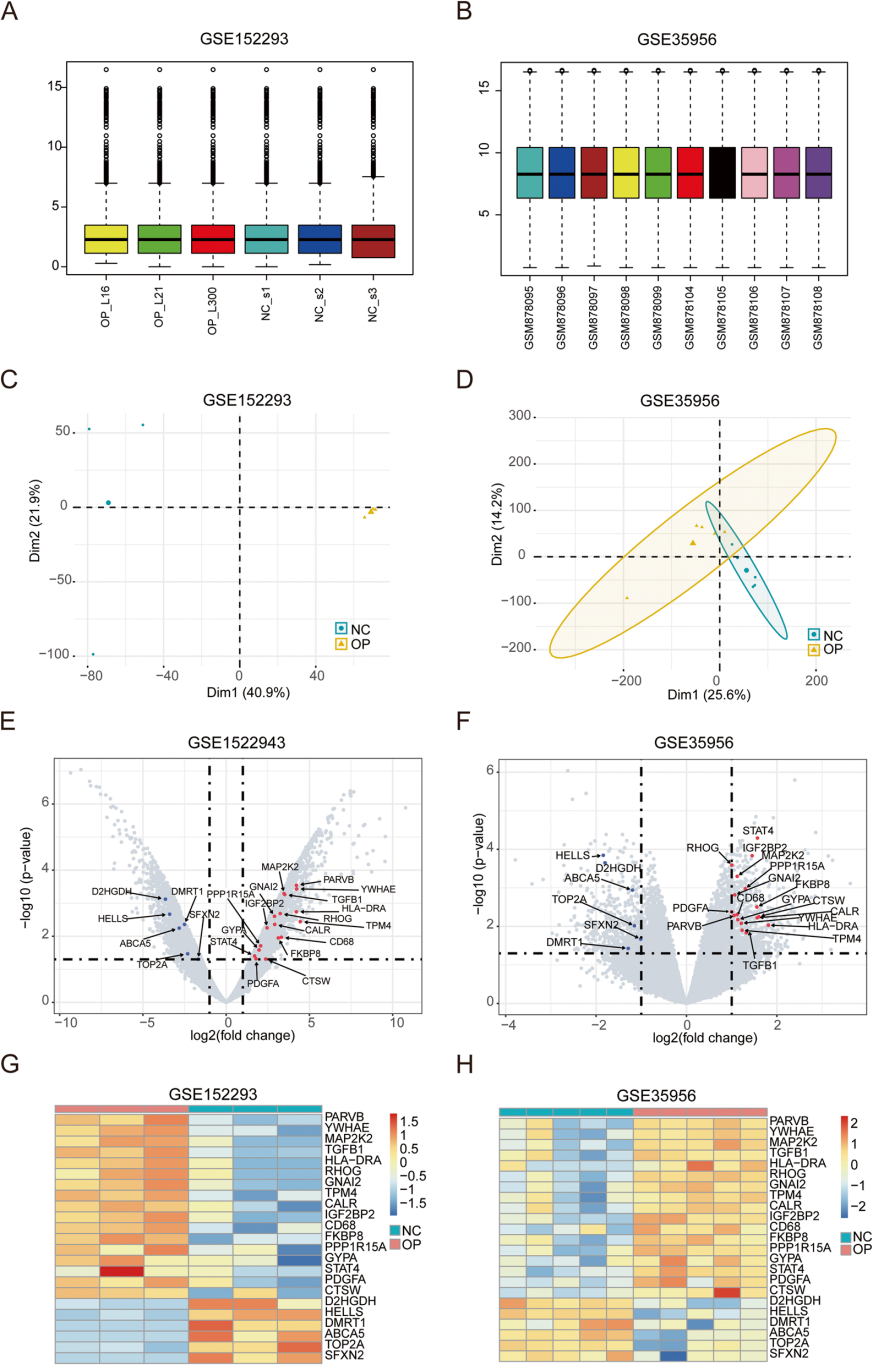
Data Pre-processing and Differential Expression Analysis. **A-B** Box plot of expression distribution between GSE152293 (**A**) and GSE35956 (**B**) samples. The x-axis represents samples, and the y-axis represents gene expression values. **C-D** PCA reduced-dimension diagrams of GSE152293 (**C**) and GSE35956 (**D**). The x-axis and y-axis represent the two reduced dimensions, and the dots in the diagrams represent samples. Healthy samples are shown in green, and osteoporosis samples are shown in yellow. **E-F** Volcano plots of differentially expressed iron metabolism-related genes for GSE152293 (**E**) and GSE35956 (**F**). The x-axis represents log2 (fold change), and the y-axis represents -log10 (*P*-value). Each dot represents a gene. Blue are downregulated iron metabolism-related genes; red are upregulated iron metabolism-related genes; gray are genes with no significant change in expressions or genes unrelated to iron metabolism. **G-H** Heatmaps of differentially expressed iron metabolism-related genes for GSE152293 (**G**) and GSE35956 (**H**). The color bars at the top represent the two groups of patients. Yellow means osteoporosis samples, and green means healthy samples. The blue blocks in the diagram represent low expressions, and the red blocks represent high expressions

Regarding the datasets of GSE152293 and GSE35956, we first examined the distribution differences between samples (Fig. 2A, B) and adopted the PCA approach to assess gene expression profiles (Fig. 2C, D). Gene expression profiles were uniformly distributed among patients, and individual differences were corrected, which was more conducive to downstream analysis. In addition, the PCA reduced-dimension results showed that normalized gene expression profiles exhibited a stronger ability to distinguish patient types.

To reveal the biological differences between osteoporosis patients and healthy controls from the transcriptome perspective, the DEGs were first analyzed. A total of 23 expressed iron metabolism-related genes (Fig. 2E-H; Table 1) were found, including 17 upregulated and 6 downregulated genes.

**Table 1:** Differentially expressed iron metabolism-related genes

Gene	Avg_logFC	avg_P.Value	Up_or_DN
PARVB	2.895533215	1.70E-04	UP
YWHAE	2.842901205	2.59E-04	UP
MAP2K2	2.237647914	3.84E-04	UP
TGFB1	2.319244929	5.24E-04	UP
HLA-DRA	2.758934647	1.44E-03	UP
RHOG	2.157490117	1.79E-03	UP
GNAI2	2.229553685	2.82E-03	UP
TPM4	2.724697241	3.91E-03	UP
CALR	2.017945594	4.67E-03	UP
IGF2BP2	1.754430584	5.40E-03	UP
CD68	2.459075994	8.16E-03	UP
FKBP8	2.346877436	8.61E-03	UP
PPP1R15A	1.604244872	1.31E-02	UP
GYPA	1.593697937	1.71E-02	UP
STAT4	1.759895514	2.44E-02	UP
PDGFA	1.509119696	3.03E-02	UP
CTSW	1.845857025	3.18E-02	UP
D2HGDH	-2.74501111	4.49E-04	DN
HELLS	-2.598041476	1.19E-03	DN
DMRT1	-1.848009625	2.76E-03	DN
ABCA5	-1.987037236	7.65E-03	DN
TOP2A	-1.663967712	2.77E-02	DN
SFXN2	-1.483849159	4.34E-02	DN

### Gene functional enrichment analysis

**Fig. 3:**
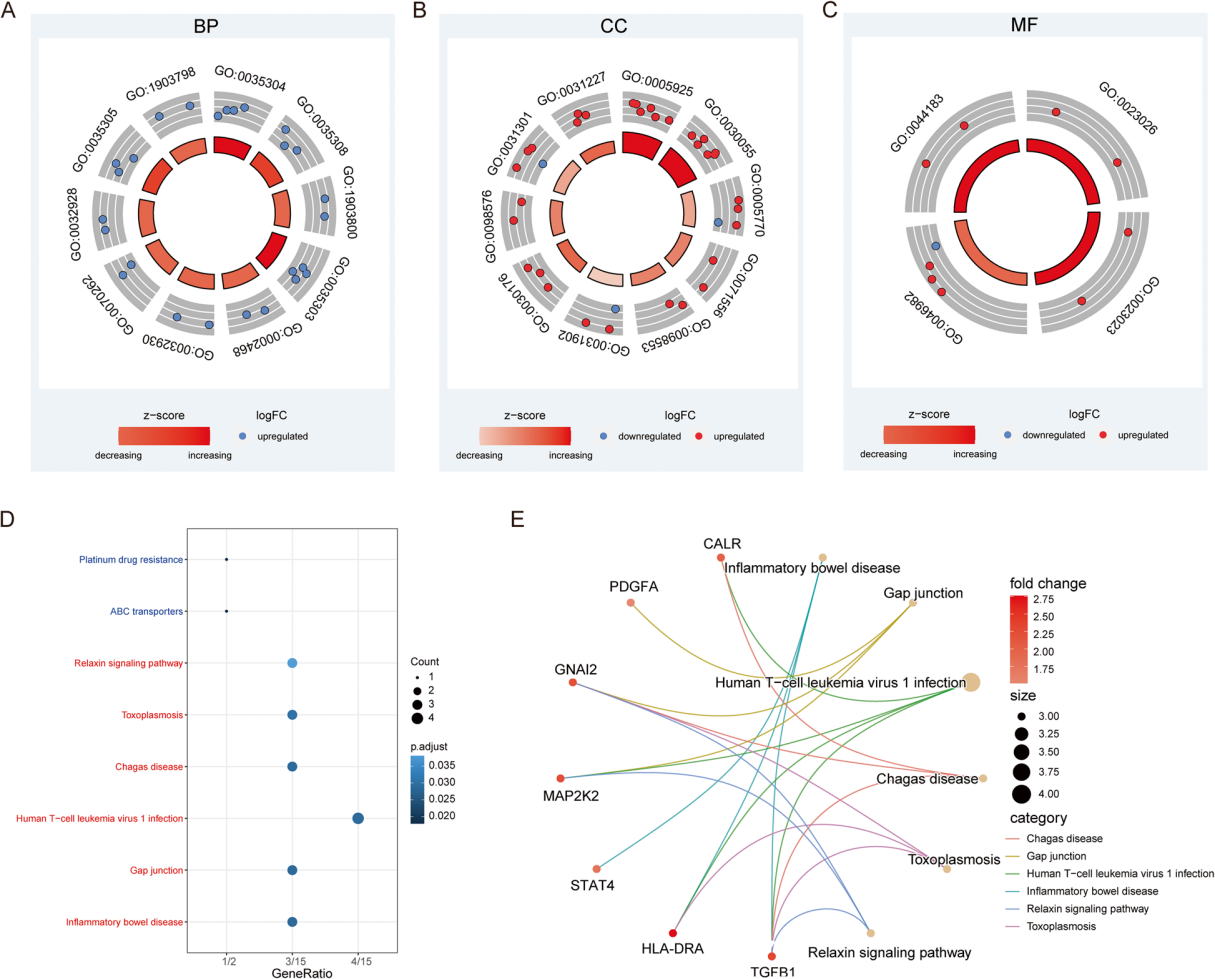
GO/KEGG Enrichment Analysis of Differentially Expressed Iron Metabolism-related Genes. **A-C** Annular diagrams of GO enrichment analysis results at the BP (**A**), CC (**B**), and MF (**C**) levels. The outmost side of the left circle is the GO term ID. The middle ring represents upregulated and downregulated genes. Each dot represents one gene that is enriched in this GO term. The color of the innermost circle represents the z-score. A gene that is closer to red has a greater upregulation degree. The length of the color bar represents the adjusted *P*-value. A longer color bar means a smaller adjusted *p*-value. **D** Bubble chart of KEGG enrichment results. The x-axis represents gene ratio; color represents adjusted *p*-value; the red labels on the y-axis represent the enrichment results of upregulated iron metabolism-related genes, and the blue labels represent the enrichment results of downregulated iron metabolism-related genes. The x-axis represents the gene ratio, i.e., the number of genes enriched in the pathway/the total number of differentially expressed genes. The y-axis represents pathways. The size of the dots represents the number of genes enriched in a pathway. The color represents the adjusted *p*-value. Dark blue represents a smaller adjusted *p*-value. **E** KEGG enrichment pathways of upregulated iron metabolism-related genes. The dot size represents a number of genes in the pathway. The pink color bar represents fold change value of genes. The number of dots represents gene entrez ID.

To further reveal the biological functions and processes affected by differentially expressed iron metabolism-related genes, GO and KEGG enrichment analysis was performed based on the expression profiles of these genes, and visualization in various forms was carried out. GO enrichment results showed that the top five BP were regulation of protein dephosphorylation, negative regulation of protein dephosphorylation, positive regulation of production of miRNAs involved in gene silencing by miRNA, regulation of dephosphorylation, and dendritic cell antigen processing and presentation (Fig. 3A; Table 2). The top five CC were focal adhesion, cell-substrate junction, late endosome, an integral component of the lumenal side of the endoplasmic reticulum membrane, and lumenal side of endoplasmic reticulum membrane (Fig. 3B; Table 2). At the MF level, genes were enriched in MHC class II protein complex binding, MHC protein complex binding, protein heterodimerization activity, and protein folding chaperone (Fig. 3C; Table 2). Notably, the most significantly enriched GO terms were upregulated compared with the early stage, suggesting that the iron metabolism-related genes in patients with osteoporosis were more active. The enriched KEGG pathways included inflammatory bowel disease, gap junction, human T-cell leukemia virus 1 infection, Chagas disease, and toxoplasmosis, all of which were related to immune functions (Fig. 3D-E; Table 2).

**Table 2:** GO and KEGG enrichment analysis

**ONTOLOGY**	**Description**	***p***.adjust
BP	regulation of protein dephosphorylation	0.027954
BP	negative regulation of protein dephosphorylation	0.032084
BP	positive regulation of production of miRNAs involved in gene silencing by miRNA	0.032084
BP	regulation of dephosphorylation	0.032084
BP	dendritic cell antigen processing and presentation	0.033901
BP	positive regulation of superoxide anion generation	0.038198
BP	peptidyl-serine dephosphorylation	0.038198
BP	regulation of superoxide anion generation	0.038198
BP	negative regulation of dephosphorylation	0.039042
BP	regulation of production of miRNAs involved in gene silencing by miRNA	0.039042
BP	regulation of production of small RNA involved in gene silencing by RNA	0.039042
BP	platelet degranulation	0.039042
BP	ERK1 and ERK2 cascade	0.039042
BP	salivary gland morphogenesis	0.039042
BP	positive regulation of gene silencing by miRNA	0.039042
BP	protein dephosphorylation	0.039042
BP	positive regulation of posttranscriptional gene silencing	0.039042
BP	salivary gland development	0.039042
BP	regulation of meiotic nuclear division	0.039042
BP	regulation of superoxide metabolic process	0.039534
BP	superoxide anion generation	0.045066
BP	macrophage derived foam cell differentiation	0.048508
BP	foam cell differentiation	0.048508
CC	focal adhesion	0.000446
CC	cell-substrate junction	0.000446
CC	late endosome	0.011145
CC	integral component of lumenal side of endoplasmic reticulum membrane	0.011223
CC	lumenal side of endoplasmic reticulum membrane	0.011223
CC	late endosome membrane	0.011223
CC	integral component of endoplasmic reticulum membrane	0.011223
CC	lumenal side of membrane	0.011223
CC	integral component of organelle membrane	0.011223
CC	intrinsic component of endoplasmic reticulum membrane	0.011223
CC	intrinsic component of organelle membrane	0.012656
CC	platelet alpha granule lumen	0.028261
CC	microvillus	0.04362
CC	platelet alpha granule	0.04362
CC	secretory granule lumen	0.04362
CC	Golgi lumen	0.04362
CC	cytoplasmic vesicle lumen	0.04362
CC	vesicle lumen	0.04362
MF	MHC class II protein complex binding	0.024483
MF	MHC protein complex binding	0.026202
MF	protein heterodimerization activity	0.026202
MF	protein folding chaperone	0.027946
**ID**	**Description**	***p*** **.adjust**
hsa05321	Inflammatory bowel disease	0.043013
hsa04540	Gap junction	0.043013
hsa05166	Human T-cell leukemia virus 1 infection	0.043013
hsa05142	Chagas disease	0.043013
KEGG enrichment analysis:		
**ID**	**Description**	***p*** **.adjust**
hsa05321	Inflammatory bowel disease	0.043013
hsa04540	Gap junction	0.043013
hsa05166	Human T-cell leukemia virus 1 infection	0.043013
hsa05142	Chagas disease	0.043013

**Fig. 4:**
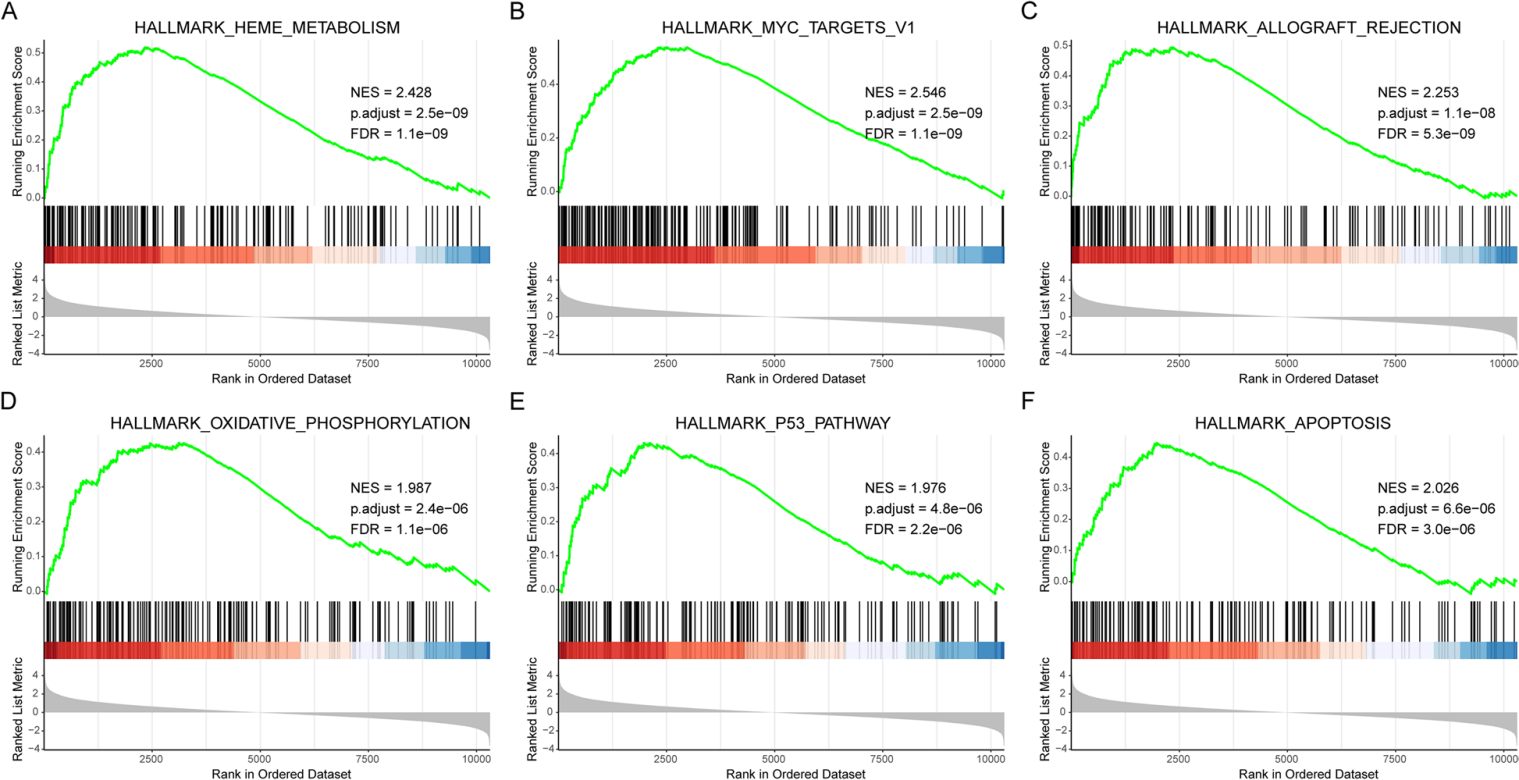
GSEA Enrichment Analysis.** A-F** Top 6 GSEA results based on genes with a mean fold change of GSE155293 and GSE35956: HALLMARK_HEME_METABOLISM (**A**), HALLMARK_MYC_TARGETS_V1 (**B**), HALLMARK_ALLOGRAFT_REJECTION (**C**), HALLMARK_OXIDATIVE_PHOSPHORYLATION (**D**), HALLMARK_P53_PATHWAY (**E**), and HALLMARK_APOPTOSIS (**F**). The x-axis represents the rank of genes in the differentially expressed gene list. The upper y-axis represents the enrichment scores, and the lower y-axis represents the logFC value. Red represents up-regulation, which indicates logFC > 0, and blue represents down-regulation, which indicates logFC < 0. The vertex of the curve on the left represents the pathway mainly enriched by upregulated genes

Based on the KEGG background gene set, enrichment analysis was performed on all DEGs using the GSEA method to further confirm the conclusion of this study’s enrichment analysis. Our results showed that the most significant top five enrichment pathways (all upregulated pathways) were heme metabolism, Myc targets v1, allograft rejection, oxidative phosphorylation, and P53 pathway (Fig. 4; Table 3).

**Table 3:** GSEA enrichment analysis

ID	NES	p.adjust
HALLMARK_HEME_METABOLISM	2.428289	2.50E-09
HALLMARK_MYC_TARGETS_V1	2.546225	2.50E-09
HALLMARK_ALLOGRAFT_REJECTION	2.253441	1.15E-08
HALLMARK_OXIDATIVE_PHOSPHORYLATION	1.987409	2.43E-06
HALLMARK_P53_PATHWAY	1.975652	4.83E-06
HALLMARK_APOPTOSIS	2.025609	6.61E-06
HALLMARK_COMPLEMENT	1.995983	6.61E-06
HALLMARK_UNFOLDED_PROTEIN_RESPONSE	2.077167	9.99E-06
HALLMARK_DNA_REPAIR	1.984705	9.99E-06
HALLMARK_REACTIVE_OXYGEN_SPECIES_PATHWAY	2.163048	2.18E-05
HALLMARK_UV_RESPONSE_UP	1.983365	4.35E-05
HALLMARK_INTERFERON_GAMMA_RESPONSE	1.81101	6.18E-05
HALLMARK_MTORC1_SIGNALING	1.82795	6.59E-05
HALLMARK_PI3K_AKT_MTOR_SIGNALING	1.933942	0.000101
HALLMARK_TNFA_SIGNALING_VIA_NFKB	1.770026	0.000286
HALLMARK_INTERFERON_ALPHA_RESPONSE	1.849241	0.000498
HALLMARK_FATTY_ACID_METABOLISM	1.747021	0.000573
HALLMARK_APICAL_JUNCTION	1.753136	0.000655
HALLMARK_HYPOXIA	1.698106	0.000866
HALLMARK_COAGULATION	1.771877	0.001165
HALLMARK_KRAS_SIGNALING_UP	1.524617	0.011802
HALLMARK_EPITHELIAL_MESENCHYMAL_TRANSITION	1.479446	0.016772
HALLMARK_TGF_BETA_SIGNALING	1.558825	0.022355
HALLMARK_ANGIOGENESIS	1.669469	0.022669
HALLMARK_ADIPOGENESIS	1.41419	0.024853
HALLMARK_ANDROGEN_RESPONSE	1.473469	0.034077

### Immune infiltration analysis

**Fig. 5:**
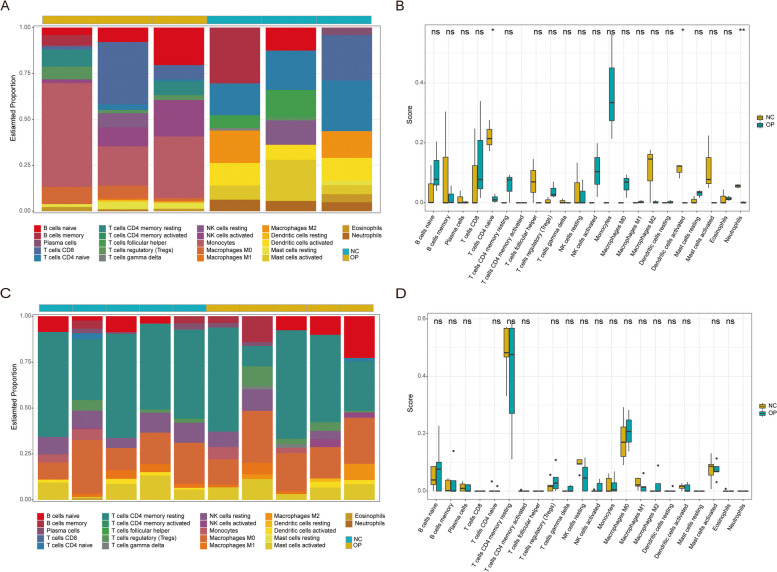
CIBERSORT Immune Filtration Assessment. **A** and **C** Stacked histograms of immune infiltration assessment for GSE152293 (**A**) and GSE35956 (**C**. The x-axis represents corresponding samples, and the y-axis represents the proportion of immune cells. Each color represents a type of immune cell. The column length represents the proportion of such immune cells in the whole. Green indicates healthy samples (NC), and yellow indicates patients with osteoporosis (OP). **B** and **D** Box diagrams of immune infiltration scoring for GSE152293 (**B**) and GSE35956 (**D**). The x-axis represents 22 types of immune cells, and the y-axis represents the infiltration level. Each color represents a patient group. The *statistical test method is the t-test. The symbols above represent the significance level of the difference.* * mean *p* < 0.05, ** means *p* = 0.01, *** means *p* = 0.001, **** means *p* = 0.0001, ns or blank means no significant difference

To compare the differences in the infiltration level of immune cells and further explore the differences in the infiltration level of immune cells between patients with osteoporosis and healthy controls, immune cell infiltration scores were calculated for all samples based on the background gene set of 22 types of immune cells in the CIBERSORT software. As shown in Fig. 5A, B, in the GSE152293 dataset, 3 cell types significantly differed between the two groups of patients, including naïve CD4 + T cells, activated dendritic cells, and neutrophils, which showed an infiltration level of almost zero in the group of patients with osteoporosis and high infiltration level in the group of healthy controls. However, there was no significant difference in the immune infiltration degree between the groups in the GSE35956 dataset (Fig. 5C-D).

**Fig. 6:**
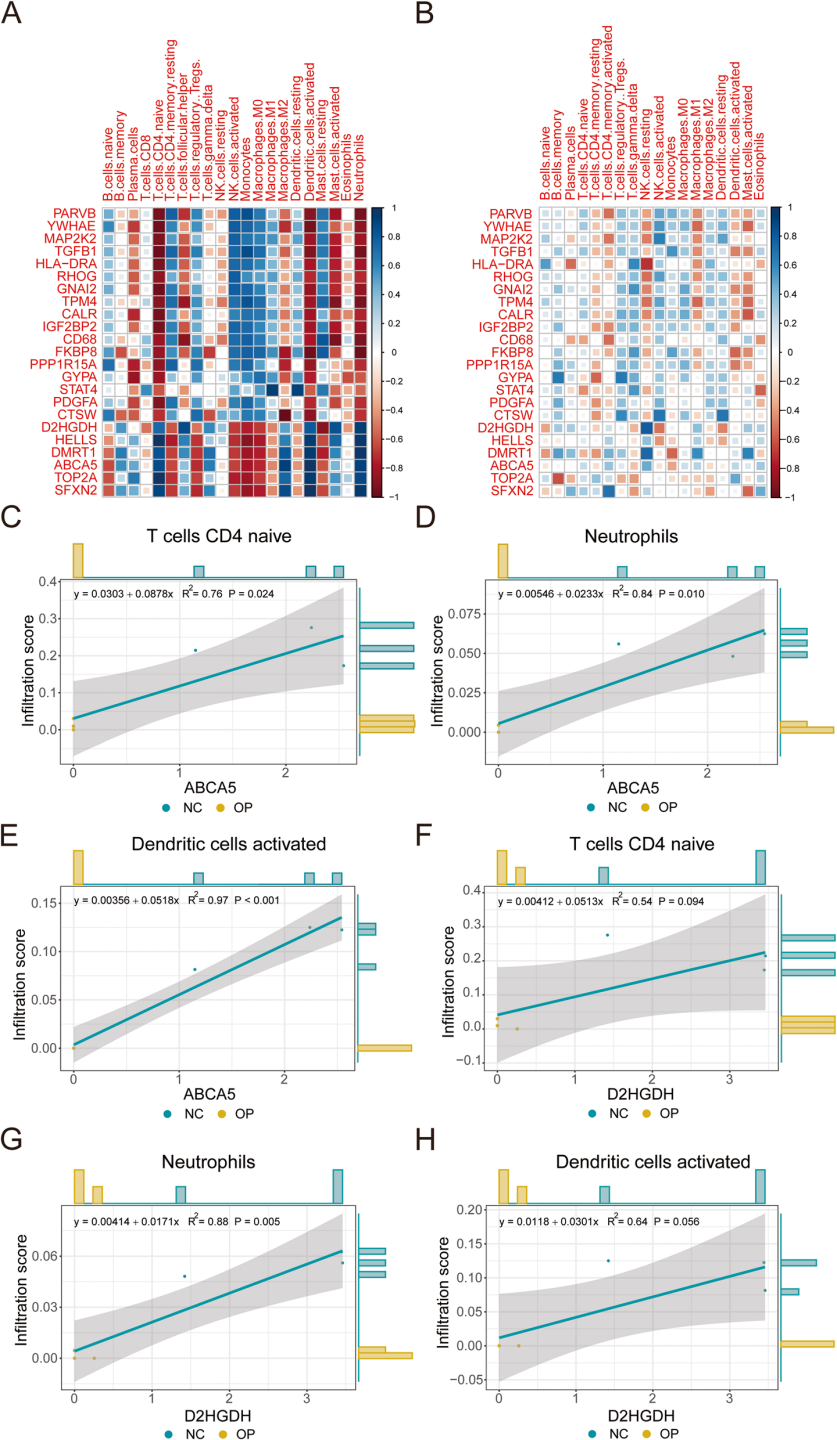
Correlation Analysis between the Expression Values of Differentially Expressed Iron Metabolism-related Genes and Immune Cells. **A** and **B** Heatmaps of Pearson correlation between the expression values of differentially expressed iron metabolism-related genes and immune cells in GSE152293 (**A**) and GSE35956 (**B**). **C-E** Scatter diagrams of Pearson correlation between ABCA5 and naïve CD4 + T cells (**C**), neutrophils (**D**), and activated dendritic cells (**E**). The x-axis represents the mean ABCA5 expression of all samples, and the y-axis represents the infiltration level of immune cells. Each dot represents a patient sample, and each color represents a patient group. The straight line in a diagram is the correlation fitting curve, the dashed parts are confidence intervals, and the outside part of the diagram is the corresponding expression histograms of samples. **F-H** Scatter diagrams of Pearson correlation between D2HGDH and naïve CD4 + T cells (**F**), neutrophils (**G**), and activated dendritic cells (**H**). The x-axis represents the mean D2HGDH expression of all samples, and the y-axis represents the infiltration level of immune cells. Each dot represents a patient sample, and each color represents a patient group. The straight line in a diagram is the correlation fitting curve, the dashed parts are confidence intervals, and the outside part of the diagram is the corresponding expression histograms of samples

To elucidate the relationship between the expressions of differentially expressed iron metabolism-related genes and the immune cell infiltration levels, scatter diagrams were created based on the expression values of the three significant differential immune cells and the differentially expressed iron metabolism-related genes, with correlation curves being fitted at the same time. The results showed that the expressions of genes, including ABCA5 and D2HGDH, were positively correlated with the infiltration degree of the three types of significantly different cells (Fig. 6), CTSW was negatively correlated with dendritic cells activated, and GNAI2 was negatively correlated with the three types of significantly different cells.

### Interaction network analysis

**Fig. 7:**
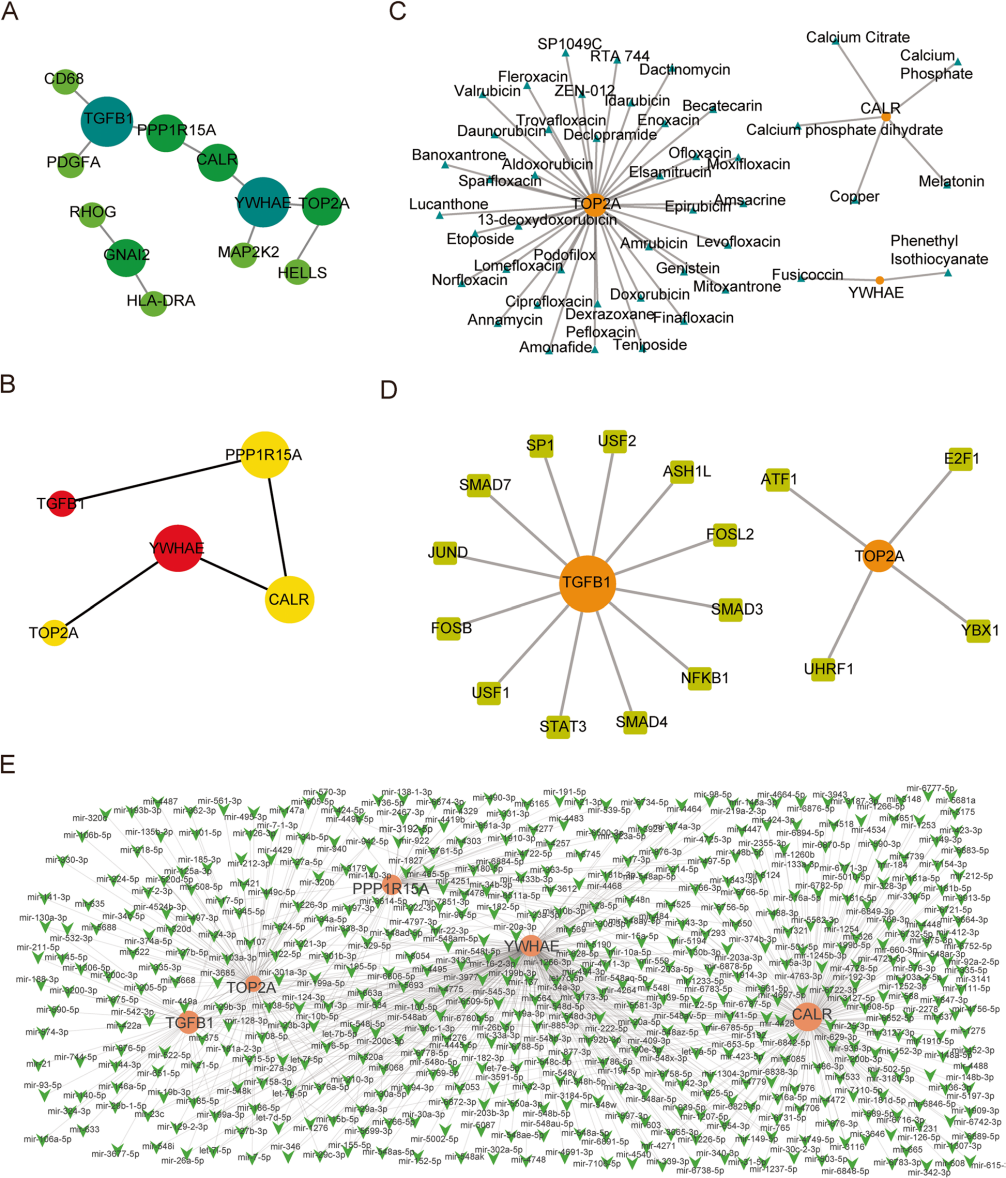
Network Analysis of Differentially Expressed Iron Metabolism-related Genes. **A** Protein-protein interaction (PPI) network of 23 differentially expressed iron metabolism-related genes. The size of dots and color represent the node degree of the network. The larger the dot, the darker the color, and the greater the node degree. **B** Top 5 hub genes based on MCC score. The color of the dots indicates MMC scoring, and the score increases gradually from yellow to red. The size of the dots means node degree. The larger the dot, the greater the node degree. **C** The hub-drug sub-network. Orange dots represent hub genes, blue triangles represent drugs, and the size of the dots represents node degree. **D** The hub-TF sub-network. Orange dots represent hub nodes, chartreuse squares represent TFs, and the size of dots represents node degree. **E** The hub-miRNA sub-network. Orange dots represent hub genes, green arrows represent miRNAs, and the size of the dots represents node degree

To further determine the relationship among differentially expressed iron metabolism-related genes and analyze the hub genes with important regulatory functions, a PPI network was constructed. The network contained 12 differentially expressed iron metabolism-related genes and 10 edges representing 10 pairs of interactions. All nodes were colored according to the size of degrees to more visually identify the hub genes in the network (Fig. 7A), after which the hub genes were mined using the cytoHubba plug-in in Cytoscape with the MCC algorithm. The nodes were scored and sorted in descending order according to the MCC results, and the top five nodes were selected as the hub nodes (Fig. 7B). These genes were: YWHAE, TGFB1, PPP1R15A, TOP2A, and CALR.

Hub genes often have an important role in biological processes, thus being more active in interacting with other biomolecules, such as miRNAs and transcription factors (TFs). In addition, they also have great potential as targets for small-molecule drugs. As a result, we predicted miRNAs and TFs to be associated with the five hub genes through the miRNet database, identified the small-molecule drugs interacting with the hub genes through the DrugBank database, and generated relevant sub-networks using the Cytoscape software. The hub-miRNA sub-network contained 705 interaction pairs and 489 miRNAs (Fig. 7C); the hub-TF sub-network contained 16 interaction pairs and 16 TFs (Fig. 7D); the hub-drug sub-network contained 45 interaction pairs and 45 small-molecule drugs (Fig. 7E).

### Validation

**Fig. 8:**
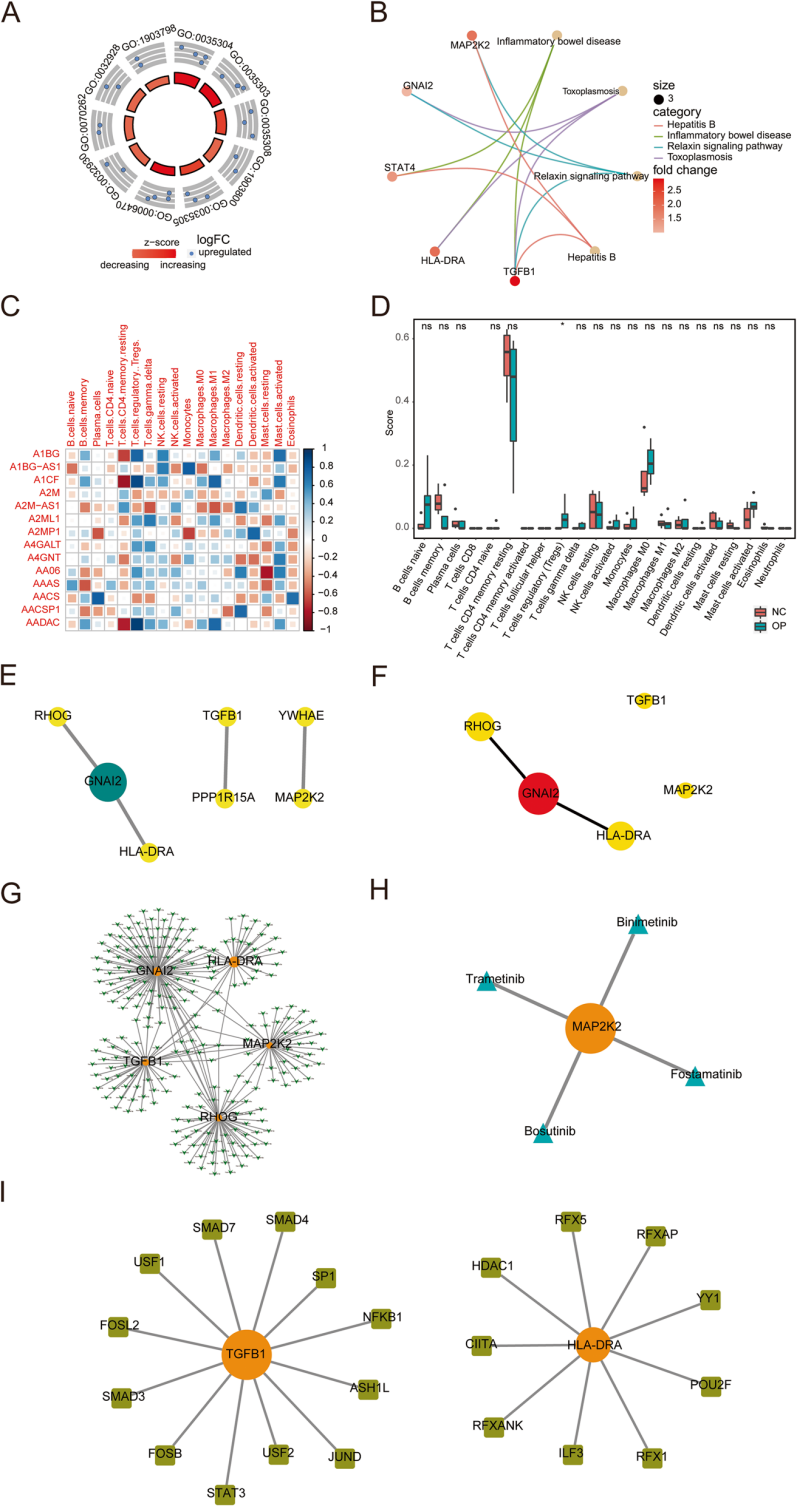
Results Validation in GSE35958 dataset. **A** GO enrichment results of differentially expressed iron-metabolism-related genes. The outmost side of the left circle is the GO term ID. The middle ring represents upregulated iron-metabolism-related genes. Each dot represents one gene that is enriched in this GO term. The color of the innermost circle represents the z-score. A gene that is closer to red has a greater upregulation degree. The length of the color bar represents the adjusted *P*-value. A longer color bar means a smaller adjusted *p*-value. **B** KEGG enrichment results of differentially expressed iron-metabolism-related genes. The dot size represents a number of genes in the pathway. The pink color bar represents fold change value of genes. The number of dots means gene entrez ID. **C** Heatmap of the Pearson correlation between immune cell infiltration levels through CIBERSORT and differentially expressed iron metabolism-related genes. Red represents negative correlation and blue represents positive correlation. **D** Differences in immune infiltration between osteoporosis and healthy group. Y-axis indicates the CIBERSORT immune infiltration results, and the x-axis indicates different immune cells. Yellow presents the osteoporosis group (OP), and green presents normal control (NC). * means *p* < 0.05, ** means *p* = 0.01, *** means *p* = 0.001, **** means *p* = 0.0001, ns or blank means no significant difference. **E** Protein-protein interaction (PPI) network of differentially expressed iron metabolism-related genes. The colors changing from yellow to green represent an increase in the degree of nodes. **F** Top five hub genes. The color of the dots represents the MCC score, and the color changing from yellow to red indicates a gradually increasing score. The size of the dots indicates the size of the degree. **G** The hub-drug sub-network. Orange dots represent hub nodes, blue triangles represent drugs, and the dots’ size indicates the degree’s size. **H** The hub-TF network. Orange dots represent hub nodes, yellowish-green squares represent TFs, and the dots’ size indicates the degree’s size. **I** The hub-miRNA sub-network. Orange dots represent hub nodes, green arrows represent miRNAs, and the dots’ size represents the degree’s size

The above analyses were validated using the validation dataset GSE35958. Data pre-processing is shown in detail in Part 1 of Supplementary Materials. After intersecting the two datasets of GSE152293 and GSE35956 with GSE35958, 14 differentially expressed iron metabolism-related genes were obtained, including 11 upregulated and 3 downregulated genes. Similarly, the GO analysis results showed that genes were enriched in pathways, such as regulation of protein dephosphorylation, negative regulation of protein dephosphorylation, and positive regulation of production of miRNAs involved in gene silencing by miRNA and regulation of dephosphorylation (Fig. 8A). The KEGG analysis showed that genes were enriched in pathways such as inflammatory bowel disease and toxoplasmosis (Fig. 8B). The GSEA results showed that genes were enriched in pathways such as heme metabolism, Myc targets v1, allograft rejection, oxidative phosphorylation, and p53 pathway (see Part 2 and Supplementary Fig. 2 of Supplementary Materials for the GSEA of the validation dataset GSE35958). The immune infiltration results showed that only regulatory T cells (Tregs) were significantly different (Fig. 8C-F). The hub nodes of the PPI network included GNAI2, RHOG, HLA-DRA, TGFB1, and MAP2K2. Figure 8G-I represent the hub-miRNA, hub-drug, and hub-TF sub-networks.

**Fig. 9:**
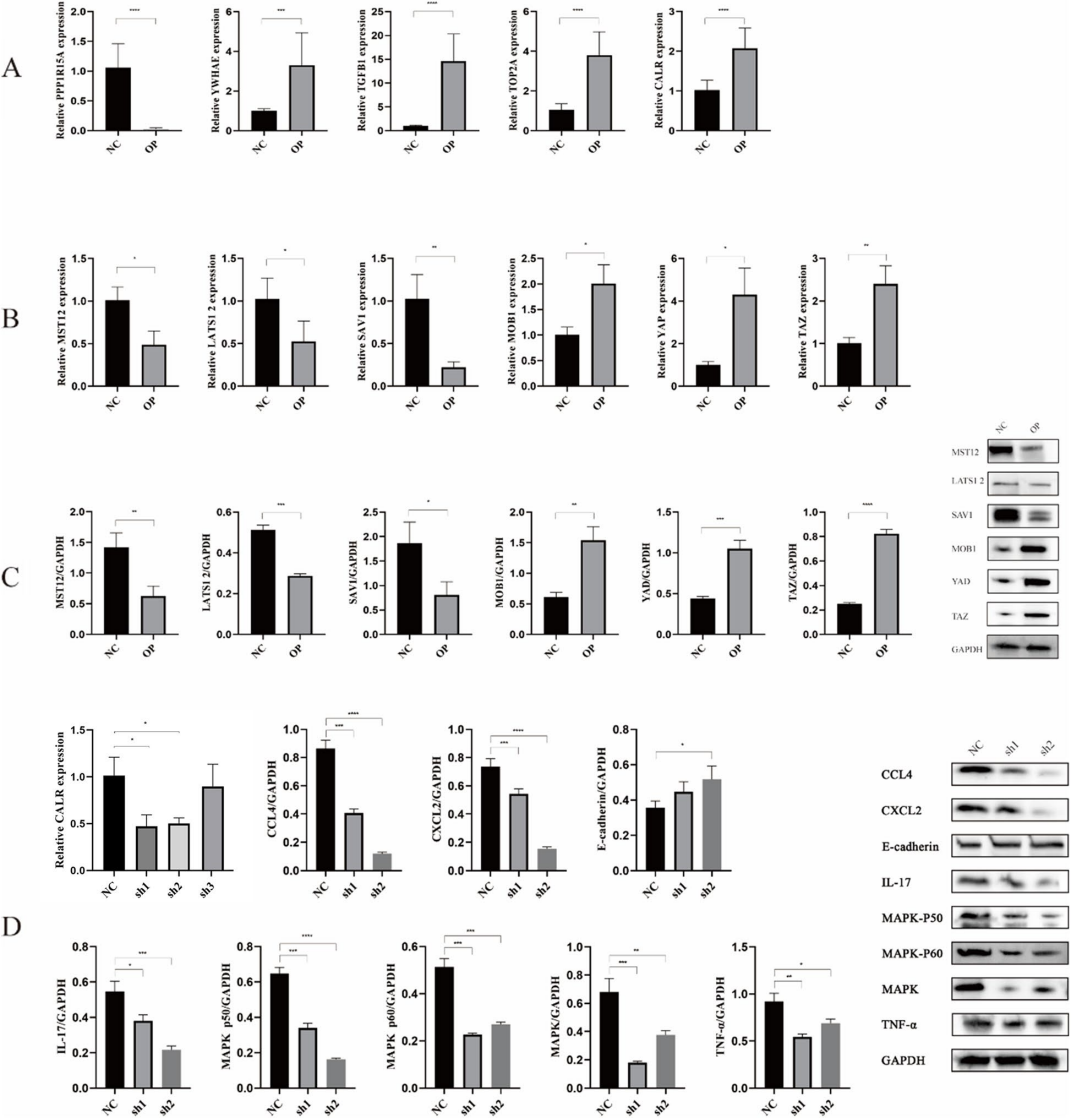
qRT-PCR and Western blotting experiment validation. **A** Hub gene expression levels in bone tissue lines were detected using qRT-PCR. **B** and **C** qRT-PCR (**B**) and Western blotting (**C**) showing the expression of related proteins and genes in the oxidative stress pathway, respectively. **D** The expression of downstream pathway-related proteins of oxidative stress pathway. *The symbols above represent the significance level of the difference.* * means < 0.05, ** means = 0.01, *** means = 0.001, **** means = 0.0001, ns mean no significant difference

The qRT-PCR method was used to verify the hub genes analyzed using bioinformatics (Fig. 9). The expression of TGF-β1, YWHAE, TOP2A and CALR increased while the expression of ppp1r15a decreased, which was similar to the bioinformatics analysis results (Fig. 9A). Many signal pathways were enriched by GSEA, with oxidative phosphorylation and the p53 pathway as the well-known signal pathways this time. Next, the oxidative stress pathway was selected to verify the expression of related proteins and genes in the pathway by qRT PCR and Western blot. The results showed that MOB1, YAP and TAZ molecules were highly expressed at the gene and protein levels (Fig. 9B, C). At the same time, we verified the expression of downstream pathway-related proteins of oxidative stress pathway, such as TNF, MAPK, IL-17, MAPK p50, MAPK p65, CCL4 and CXCL2 (Fig. 9D).

## Discussion

Recent studies have suggested that osteoporosis is closely related to iron metabolism, and that disorders of iron metabolism affect the dynamic balance of bones [2, 3]. Iron overload promotes osteoclast differentiation and osteoblast apoptosis and inhibits the proliferation and differentiation of osteoblasts. Additionally, iron deficiency affects collagen synthesis and vitamin D metabolism [4]. However, little research has been conducted at the genetic level. The development of high-throughput sequencing technology has made it possible for us to understand and study the mechanism of diseases at the genetic level, thus facilitating the identification of new biomarkers [32, 33].

In this study, 23 differentially expressed iron metabolism-related genes, including 17 upregulated and 6 downregulated genes, were identified by downloading online databases, analyzing the DEGs in the osteoporosis datasets, and intersecting these genes with iron metabolism-related genes. Next, the GO and KEGG enrichment analyses were performed. In the GO enrichment analysis, BP results were related to the immune process involved by dendritic cells. This indicated that, compared with healthy people, patients with osteoporosis have a significant correlation with the antigen presentation of dendritic cells, which is related to iron metabolism [34]. It is worth mentioning that most of the most significantly enriched GO terms were upregulated compared with the early stage, thus indicating that the iron metabolism-related genes in osteoporosis patients were more active, which is also in line with existing literature [35]. In addition, many pathways in the KEGG enrichment analysis were associated with immune function. In particular, under oxidative stress conditions, the gap junction pathway has a critical role in bone remodeling, mechanical transduction, and osteocyte survival and is closely associated with disuse osteoporosis [36–38].

The top five significant pathways found in this study were heme metabolism, Myc targets v1, allograft rejection, oxidative phosphorylation, and p53 pathway, all being upregulated pathways. It has been reported that heme can inhibit cell death via HO-1 and caspase-3 mediation, and HO-1 induction can be used to treat glucocorticoid-related osteonecrosis and osteoporosis [39]; this process is closely associated with the heme metabolism pathway. Oxidative phosphorylation is closely related to osteoporosis, and it has been shown that its main biological behavior in the formation of osteoporosis is to promote bone loss [40, 41]. It is generally known that the p53 pathway is an important signal pathway for osteoblast differentiation [42], and the stability regulation by p53 is important in osteoblast differentiation [43]. Meanwhile, the downregulated expression of p53 may be a potential marker for drug therapy for osteoporosis [44, 45].

The immune system is intricately involved in bone physiology as well as pathologies. The immune microenvironment is a loaded, integrated system that consists of immune cells, inflammatory cells, fibroblasts, interstitial tissues, and various cytokines and chemokines. The infiltration analysis of immune cells in the tissue has an important directive function in disease research, treatment, and prognosis prediction [46]. Therefore, the immune system’s role in skeletal pathophysiology is becoming increasingly significant. In fact, a new research field called bone immunology has emerged. Imbalance between regulatory T cells and T helper 17 cells cause immune cells and fibroblasts to interact, which can aggravate inflammation, promote the occurrence of bone destruction [47]. Immune cells, such as t lymphocyte subsets (Th17), trigger immune disorders that produce inflammation that adversely affects bone integrity [48]. Immune cells also contribute to osteoporosis by producing pro-inflammatory mediators and modulating the RANK/RANKL/OPG axis [49].

In this study, we found that three types of immune cells in the GSE152293 dataset, including naïve CD4 + T cells, activated dendritic cells, and neutrophils significantly differed between the two groups of patients. T cells have a central role in the interaction pathway of osteoclast formation, osteoclast formation, and bone remodeling [50]. Activated dendritic cells contribute to inflammation-mediated osteoclastogenesis and take part in inflammatory bone disease, which can activate T-cells by acting as APCs. Also, activated T-cells produce cytokines and soluble factors that drive bone remodeling [51], while neutrophils are involved in the pathophysiology of various diseases, including inflammation-mediated bone loss. Moreover, neutrophils can produce chemokines to recruit pro-osteoporotic cells such as Th17 [52]. Unfortunately, the same result was not found in the dataset GSE35956, which may be due to the differences between samples in different datasets.

To further identify the relationship among differentially expressed iron metabolism-related genes and analyze the few hub genes with important regulatory functions, we constructed a PPI network, carried out the secondary treatment, and further analyzed the network properties based on the intuitive, visualized network. The identified hub genes were TGF-β1, YWHAE, PPP1R15A, TOP2A, and CALR. Numerous studies have reported TGF-β1 to be closely associated with the occurrence and development of osteoporosis. TGF-β1 is a rich protein in the bone matrix that is stored as an inactive precursor before bone absorption begins, and increased TGF-β1 levels can promote bone formation and metabolism [53]. Additionally, TGF-β1 can downregulate the expressions of NFATc1 by blocking the nuclear translocation of NF-κB, thereby regulating osteoclast formation induced by human RANKL [54]. YWHAE, as a molecular scaffold, participates in many biological processes such as cell adhesion, cell cycle regulation, signal transduction, and malignant transformation and is closely associated with multiple diseases. Also, existing research shows YWHAE is mostly related to tumor diseases [55]; yet, its role in osteoporosis remains unclear. Previous research has confirmed that TOP2A has a positive regulatory role in bone formation metabolism [56]. CALR may interact with calcium phosphate to regulate multiple signaling pathways to treat OP [57].

Hub genes often have important roles in biological processes, thus being more active in interacting with other biomolecules, such as miRNAs and TFs. In addition, they also have great potential as targets for small-molecule drugs. As a result, we predicted miRNAs and TFs to be associated with the five hub genes through the miRNet database and identified the small-molecule drugs interacting with the hub genes through the DrugBank database. Calcium citrate, calcium phosphate, and calcium phosphate dehydrate are known compounds that are closely related to osteoporosis and are widely applied in treating patients with osteoporosis [57, 58]. However, further exploration of the function of the above drugs or molecular compounds in osteoporosis and its complications as potential treatment targets is needed.

Bioinformatics analysis is the inferential result of various statistical calculations based on chip or sequencing data. The obtained results have certain reliability but still need experimental verification. Firstly, our analysis data were from a public database, and failing to merge datasets from the same chip platform may result in batch effects. Secondly, due to the limited dataset for this disease, we chose the dataset from the different dataset platforms considering factors such as dataset quality, sample size, organizational source, species, and similar, which may cause deviation to the follow-up analysis. Thirdly, it is best to use multiple similar methods (such as xCELL and TIMER) for immune infiltration analysis to replicate and experimentally verify. These are all areas that need improvement in our future research, as this may result in higher accuracy.

## Conclusion

Collectively, through the comprehensive bioinformatics analysis, we identified a group of target genes that might be related to the treatment of osteoporosis, as well as biological pathways that might lead to changes in bone mineral density. We identified DEGs of iron metabolism and osteoporosis, analyzed the potential regulatory mechanism, and further determined the hub genes. These findings further elucidate the unique role of iron metabolism in the occurrence and development of osteoporosis and explore potential treatment targets and biomarkers. In addition, this study can provide ideas and assumptions for subsequent research.

### Data availability statement

Data are available in a public, open access repository. Data are available on reasonable request. All data relevant to the study are included in the article or uploaded as supplemental information. The datasets for this study can be found in the GEO (https://www.ncbi.nlm.nih.gov/ ; Accession numbers: GSE152293, GSE35956, GSE35958), ferroptosis phenotype database FerrDb (http://www.zhounan.org/ferrdb), Reactome database (https://reactome.org/), AmiGo2 database (http://amigo.geneontology.org/amigo/landing) and the GeneCards database (https://www.genecards.org).

### Supplementary Information

**Additional file 1: Supplementary Figure 1.** Point plots of Pearson correlations among samples in GSE152293 (A), GSE35956 (B), and GSE25958 (C) datasets. **Supplementary Figure 2.** Verification result of GSE56815 data set. **Supplementary Figure 3.*** Data Pre-processing for the Validation Dataset GSE35958*.**Supplementary Figure 4.** GSEA Results for the Validation Dataset GSE35958. **Supplementary Figure 5.** ROC Analysis. **Supplementary Figure 6.** Full-length of western blotting showing the expression of related proteins and genes in oxidative stress pathway (Fig 9-C)

## Data Availability

The datasets analyzed during the current study are available in the GEO repository (https://www.ncbi.nlm.nih.gov/; Accession numbers:GSE152293, GSE35956, GSE35958), ferroptosis phenotype database FerrDb (http://www.zhounan.org/ferrdb), Reactome database (https://reactome.org/), AmiGo2 database (http://amigo.geneontology.org/amigo/landing) and the GeneCards database (https://www.genecards.org).

## Abbreviations

DEGs: Differentially expressed genes
GEO: Gene Expression Omnibus
IRG: Iron metabolism-related genes
GO: Gene Ontology
BP: Biological process
MF: Molecular function
CC: Cellular component
KEGG: Kyoto Encyclopedia of Genes and Genomes
GSEA: Gene Set Enrichment Analysis
PPI: Protein-protein interaction
AUC: Area under the curve
DXA: Dual X-ray absorptiometry

## References

[1] An J, Yang H, Zhang Q, Liu C, Zhao J, Zhang L (2016). Natural products for treatment of osteoporosis: the effects and mechanisms on promoting osteoblast-mediated bone formation. Life Sci.

[2] Lewiecki EM (2020). Romosozumab, clinical trials, and real-world care of patients with osteoporosis. Annals of Translational Medicine.

[3] Bo L, Liu Z, Zhong Y, Huang J, Chen B, Wang H (2016). Iron Deficiency anemia’s effect on bone formation in zebrafish mutant. Biochem Biophys Res Commun.

[4] Che J, Yang J, Zhao B, Zhang G, Wang L, Peng S (2019). The effect of abnormal Iron metabolism on osteoporosis. Biol Trace Elem Res.

[5] Liu P, Wang W, Li Z, Li Y, Yu X, Tu J (2022). Ferroptosis: a New Regulatory mechanism in osteoporosis. Oxidative Med Cell Longev.

[6] Jomova K, Valko M (2011). Advances in metal-induced oxidative stress and human Disease. Toxicology.

[7] Teng Z, Zhu Y, Zhang X, Teng Y, Lu S (2020). Osteoporosis is characterized by altered expression of Exosomal Long non-coding RNAs. Front Genet.

[8] Benisch P, Schilling T, Klein-Hitpass L, Frey SP, Seefried L, Raaijmakers N (2012). The transcriptional profile of mesenchymal stem cell populations in primary osteoporosis is distinct and shows overexpression of osteogenic inhibitors. PLoS ONE.

[9] Barrett T, Wilhite SE, Ledoux P, Evangelista C, Kim IF, Tomashevsky M (2013). NCBI GEO: archive for functional genomics data sets–update. Nucleic Acids Res.

[10] Davis S, Meltzer PS (2007). GEOquery: a bridge between the Gene expression Omnibus (GEO) and BioConductor. Bioinformatics.

[11] Smyth GK (2005). Limma: linear models for microarray data. Bioinformatics and computational biology solutions using R and Bioconductor.

[12] Lê S, Josse J, Husson F (2008). FactoMineR: an R package for multivariate analysis. J Stat Softw..

[13] Zhou N, Bao J (2020). FerrDb: a manually curated resource for regulators and markers of ferroptosis and ferroptosis-disease associations. Database: The Journal of Biological Databases and Curation.

[14] Jassal B, Matthews L, Viteri G, Gong C, Lorente P, Fabregat A (2020). The reactome pathway knowledgebase. Nucleic Acids Res.

[15] Stelzer G, Rosen N, Plaschkes I, Zimmerman S, Twik M, Fishilevich S, et al. The GeneCards Suite: From Gene Data Mining to Disease Genome Sequence Analyses. Curr Protoc Bioinformatics. 2016;54:1.30.1-1.30.33. 10.1002/cpbi.5. 10.1002/cpbi.5 27322403

[16] Wickham H (2011). ggplot2. Wiley interdisciplinary reviews: computational statistics..

[17] Kolde R, Vilo J (2015). GO summaries: an R Package for Visual Functional Annotation of Experimental Data. F1000Research.

[18] Harris MA, Clark J, Ireland A, Lomax J, Ashburner M, Foulger R (2004). The Gene Ontology (GO) database and informatics resource. Nucleic Acids Res.

[19] Kanehisa M, Goto S (2000). KEGG: kyoto encyclopedia of genes and genomes. Nucleic Acids Res.

[20] Wu T, Hu E, Xu S, Chen M, Guo P, Dai Z (2021). clusterProfiler 4.0: a universal enrichment tool for interpreting omics data.

[21] Walter W, Sánchez-Cabo F, Ricote M (2015). GOplot: an R package for visually combining expression data with functional analysis: Fig. 1. Bioinformatics.

[22] Subramanian A, Tamayo P, Mootha VK, Mukherjee S, Ebert BL, Gillette MA (2005). Gene set enrichment analysis: a knowledge-based approach for interpreting genome-wide expression profiles. Proc Natl Acad Sci USA.

[23] Liberzon A, Birger C, Thorvaldsdóttir H, Ghandi M, Mesirov JP, Tamayo P (2015). The Molecular signatures database (MSigDB) hallmark gene set collection. Cell Syst.

[24] Steen CB, Liu CL, Alizadeh AA, Newman AM (2020). Profiling cell type abundance and expression in bulk tissues with CIBERSORTx. (Clifton NJ).

[25] Ahlmann-Eltze C, Patil I. ggsignif: R Package for Displaying Significance Brackets for “ggplot2.” In.: Center for Open Science; 2021.

[26] von Mering C, Huynen M, Jaeggi D, Schmidt S, Bork P, Snel B (2003). STRING: a database of predicted functional associations between proteins. Nucleic Acids Res.

[27] Shannon P, Markiel A, Ozier O, Baliga NS, Wang JT, Ramage D (2003). Cytoscape: a software environment for integrated models of biomolecular interaction networks. Genome Res.

[28] Chin C, Chen S, Wu H, Ho C, Ko M, Lin C (2014). cytoHubba: identifying hub objects and sub-networks from complex interactome. BMC Syst Biol.

[29] Chang L, Zhou G, Soufan O, Xia J (2020). miRNet 2.0: network-based visual analytics for miRNA functional analysis and systems biology. Nucleic Acids Res.

[30] Wishart DS, Knox C, Guo AC, Cheng D, Shrivastava S, Tzur D (2008). DrugBank: a knowledgebase for Drugs, drug actions and drug targets. Nucleic Acids Res.

[31] Robin X, Turck N, Hainard A, Tiberti N, Lisacek F, Sanchez J-C (2011). pROC: an open-source package for R and S + to analyze and compare ROC curves. BMC Bioinformatics.

[32] Deng Y, Ren E, Yuan W, Zhang G, Wu Z, Xie Q (2020). GRB10 and E2F3 as diagnostic markers of Osteoarthritis and their correlation with Immune Infiltration. Diagnostics (Basel Switzerland).

[33] Tian Z, He W, Tang J, Liao X, Yang Q, Wu Y (2020). Identification of important modules and biomarkers in Breast Cancer based on WGCNA. OncoTargets and Therapy.

[34] Yan H, Zou T, Tuo Q, Xu S, Li H, Belaidi AA (2021). Ferroptosis: mechanisms and links with Diseases. Signal Transduct Target Therapy.

[35] van Swelm RPL, Wetzels JFM, Swinkels DW (2019). The multifaceted role of iron in renal health and Disease. Nat Rev Nephrol.

[36] Hua R, Zhang J, Riquelme MA, Jiang JX (2021). Connexin Gap Junctions and hemichannels Link oxidative stress to skeletal physiology and Pathology. Curr Osteoporos Rep.

[37] Rolvien T, Amling M. Disuse osteoporosis: clinical and mechanistic insights. Calcified Tissue International; 2021. 10.1007/s00223-021-00836-1 PMC9013332 33738515

[38] Zhang D, Li X, Pi C, Cai L, Liu Y, Du W (2020). Osteoporosis-decreased extracellular matrix stiffness impairs connexin 43-mediated gap junction intercellular communication in osteocytes. Acta Biochim Biophys Sin.

[39] Yamamoto H, Saito M, Goto T, Ueshima K, Ishida M, Hayashi S (2019). Heme oxygenase-1 prevents glucocorticoid and hypoxia-induced apoptosis and necrosis of osteocyte-like cells. Med Mol Morphol.

[40] Mo Y, Lai W, Zhong Y, Hu Z, You M, Du M (2021). TXNIP contributes to bone loss via promoting the mitochondrial oxidative phosphorylation during glucocorticoid-induced osteoporosis. Life Sci.

[41] Kim H, Ponte F, Nookaew I, Ucer Ozgurel S, Marques-Carvalho A, Iyer S (2020). Estrogens decrease osteoclast number by attenuating mitochondria oxidative phosphorylation and ATP production in early osteoclast precursors. Sci Rep.

[42] Wang M, Huan Y, Li X, Li J, Lv G (2021). RUNX3 derived hsa_circ_0005752 accelerates the osteogenic differentiation of adipose-derived stem cells via the miR-496/MDM2-p53 pathway. Regenerative Therapy.

[43] Yang Y, Li C, Wang J, Huang X, Yuan Y, Hu J (2019). Ubiquitylomes Analysis of the whole blood in postmenopausal osteoporosis patients and healthy Postmenopausal women. Orthop Surg.

[44] Yu T, You X, Zhou H, Kang A, He W, Li Z (2020). p53 plays a central role in the development of osteoporosis. Aging.

[45] Yu T, Wu Q, You X, Zhou H, Xu S, He W (2020). Tomatidine alleviates osteoporosis by downregulation of p53. Med Sci Monitor: Int Med J Experimental Clin Res.

[46] Saxena Y, Routh S, Mukhopadhaya A, Immunoporosis (2021). Role of Innate Immune cells in osteoporosis. Front Immunol.

[47] Arron JR, Choi Y (2000). Bone versus immune system. Nature.

[48] Ahmad SS, Ahmed F, Ali R, Ghoneim MM, Alshehri S, Najmi AK (2022). Immunology of osteoporosis: relevance of inflammatory targets for the development of novel interventions. Immunotherapy.

[49] Komatsu N, Takayanagi H (2022). Mechanisms of joint destruction in rheumatoid arthritis - immune cell-fibroblast-bone interactions. Nat Rev Rheumatol.

[50] Kumar G, Roger P-M (2019). From crosstalk between Immune and Bone cells to bone Erosion in Infection. Int J Mol Sci.

[51] Maitra R, Follenzi A, Yaghoobian A, Montagna C, Merlin S, Cannizzo ES (2010). Dendritic cell-mediated in vivo bone resorption. J Immunol.

[52] Tu M, Han K, Lan Y, Chang K, Lai C, Staniczek T (2021). Association of TGF-β1 and IL-10 gene polymorphisms with osteoporosis in a study of Taiwanese osteoporotic patients. Genes.

[53] Tokunaga T, Mokuda S, Kohno H, Yukawa K, Kuranobu T, Oi K (2020). TGFβ1 regulates human RANKL-Induced Osteoclastogenesis via suppression of NFATc1 expression. Int J Mol Sci.

[54] Yang Y, Lee Y, Wang Y, Wang C, Hou M, Yuan SF (2019). YWHAE promotes proliferation, metastasis, and chemoresistance in breast cancer cells. Kaohsiung J Med Sci..

[55] Chen J, Chen L, Hua J, Song W (2021). Long-term dynamic compression enhancement TGF-β3-induced chondrogenesis in bovine stem cells: a gene expression analysis. BMC Genomic data.

[56] Yang Z, Yuan ZZ, Ma XL (2021). Network Pharmacology-based strategy and molecular docking to explore the potential mechanism of Jintiange Capsule for treating osteoporosis. Evid Based Complement Alternat Med.

[57] Palermo A, Naciu AM, Tabacco G, Manfrini S, Trimboli P, Vescini F (2019). Calcium citrate: from biochemistry and physiology to clinical applications. Reviews in Endocrine and Metabolic Disorders.

[58] Gómez JMQ, Rubió JB, Curiel MD, Pérez AD (2011). Calcium citrate and Vitamin D in the treatment of osteoporosis. Clin Drug Investig.

