# Supplementary material for: Identification and validation of iron metabolism genes in osteoporosis
Source: BMC Med Genomics. 2024 Jan 2;17:5. doi: 10.1186/s12920-023-01779-2 (PMC10762978; doi:10.1186/s12920-023-01779-2)
Supplement: Supplementary file 1 — Additional file 1: Supplementary Figure 1. Point plots of Pearson correlations among samples in GSE152293 (A), GSE35956 (B), and GSE25958 (C) datasets. Supplementary Figure 2. Verification result of GSE56815 data set. Supplementary Figure 3. Data Pre-processing for the Validation Dataset GSE35958.Supplementary Figure 4. GSEA Results for the Validation Dataset GSE35958. Supplementary Figure 5. ROC Analysis. Supplementary Figure 6. Full-length of western blotting showing the expression of related proteins and genes in oxidative stress pathway (Fig 9-C) [file 12920_2023_1779_MOESM1_ESM.pdf]

Identification and Validation of Iron Metabolism Genes in Osteoporosis

Running title: Iron Metabolism and Osteoporosis

Zutao Li¶, Jiangbo Xu¶, Shouyin Shi, Youlin Weng, Bin Guo, Lixin Che, Jungang Sun*

Department of Orthopedic Trauma, People's Hospital of Xinjiang Uygur Autonomous Region, Urumqi, Xinjiang, 830001, China

¶These authors have contributed equally to this work

*** Correspondence**

Jungang Sun

Department of Orthopedic Trauma, People's Hospital of Xinjiang Uygur Autonomous Region, Urumqi, Xinjiang, 830001, China

E-mail: 710999438@qq.com

Tel: +86-15739579385

Supplementary Materials 1

Dot plot to show correlations between samples on Supplementary Materials

Firstly, the cor () function of the stats (v4.3.2) package in R language was used to calculate the Pearson correlation between samples. Then ggplot2 (v3.3.5) [1] was used to plot Pearson correlations among samples in the GSE152293 (SF 1A), GSE35956 (SF 1B), and GSE25958 (SF 1C) datasets.

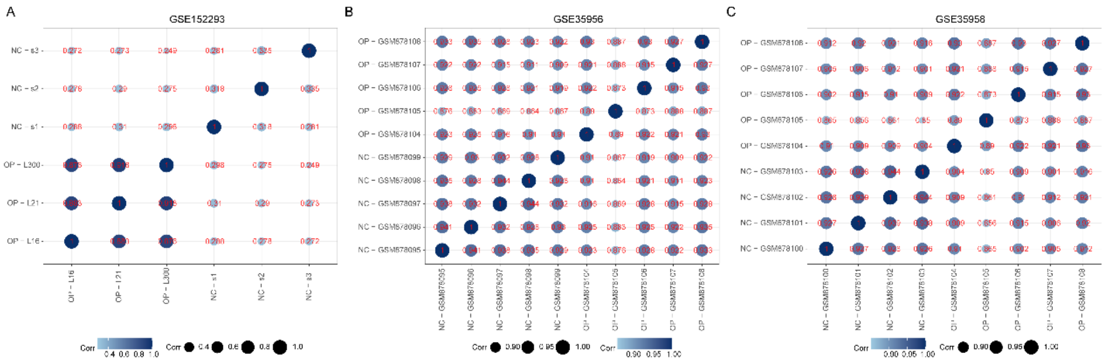

Supplementary Figure **1**: Point plots of Pearson correlations among samples in GSE152293 (A), GSE35956 (B), and GSE25958 (C) datasets.

Analysis for GSE56815 and added it on Supplementary Materials

GEO database (https://www.ncbi.nlm.nih.gov/geo/) [2] was used to download the GSE56815 osteoporosis clinical information, relevant data and the corresponding human sample sources, measured before and after menopause, reflecting high and low bone mineral density in blood mononuclear cells of the female. The sequencing platforms were GPL96 ([HG-U133A] Affymetrix Human Genome U133A Array). A total of 80 patients with GSE56815 were enrolled, including 40 patients in the low bone mineral density group (osteoporosis patients) and 40 patients in the high bone mineral density group (normal control group). GSE56815 was used as the verification set to verify the results. First, the limma package (v3.48.3) [3] was used for the normalization between samples, and the box diagram was used to visualize the expression between samples (SF 2A). Then, to see the distribution of all samples, the R package

FactoMineR (v2.4) [4] was used for Principal Component Analysis (PCA) and visualization (SF 2B).

In order to reveal the differences in gene expression between osteoporosis and normal controls (especially the differences in the expression of genes related to iron metabolism), we conducted an inter-group differential gene expression analysis. The r package was used to analyze the differentially expressed limma (v3.48.3) [3], | log2 fold change | | (log2FC |) > = 1 and P value < 0.05 set threshold for differences in the gene, log2FC > 1 and P value < 0.05 were differentially expressed genes with up-regulated expression, while log2FC < -1 and P value < 0.05 were differentially expressed genes with down-regulated expression. Visualization of volcano map using R package ggplot2 (v3.3.5) [1] (SF 2C). Results showed no significant differences in any of the genes. Subsequently, we examined 23 iron metabolization-related genes that differed in GSE152293 and GSE35956 (PARVB, YWHAE, MAP2K2, TGFB1, HLA-DRA, RHOG, GNAI2, TPM4, CALR, IGF2BP2, CD68 FKBP8, PPP1R15A, GYPA, STAT4, PDGFA, CTSW, D2HGDH, HELLS, DMRT1, BCA5, TOP2A, and XN2) in the verification set GSE56815, using R package pheatmap (v1.0.12) [5] to draw heatmap (SF 2D). There were no expressions of D2HGDH and SFXN2 in the GSE56815 data set. The Heatmap shows that the expression of 21 iron metabolism-related genes did not differ between the osteoporotic and healthy controls in the gse56815 dataset.

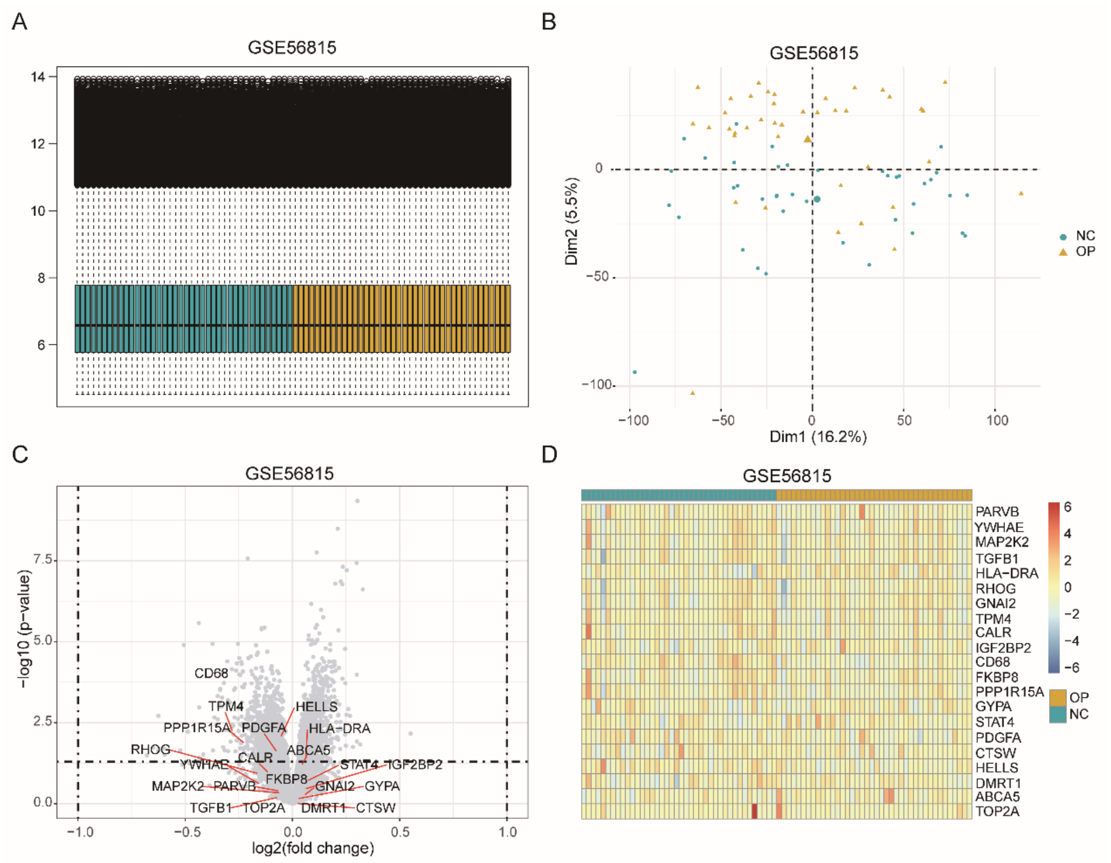

Supplementary Figure 2. Verification result of GSE56815 data set.

Data pre-processing for the validation dataset GSE35958

With respect to the validation dataset GSE35958, we first investigated the distribution differences of data between samples (Supplementary Figure 1A), and the PCA approach was adopted to view the extent to which gene expression profiles distinguished patient types (Supplementary Figure 1 B). As shown, gene expression profiles were uniformly distributed among patients, and individual differences in patients were corrected, which was more conducive to downstream analysis. In addition, the PCA reduced-dimension results showed that normalized gene expression profiles exhibited a stronger ability to distinguish patient types.

To reveal the biological differences between patients with osteoporosis and healthy people from the transcriptome perspective, *the differential expressions of genes were analyzed based on this grouping label. Then, after screening by a statistically significant threshold, the differentially expressed genes in the validation dataset and differentially expressed iron metabolism-related genes were* intersected. Only 14 genes,

including 11 upregulated and three downregulated genes, were differentially expressed iron metabolism-related genes in the three datasets (Supplementary Figure 1C-D).

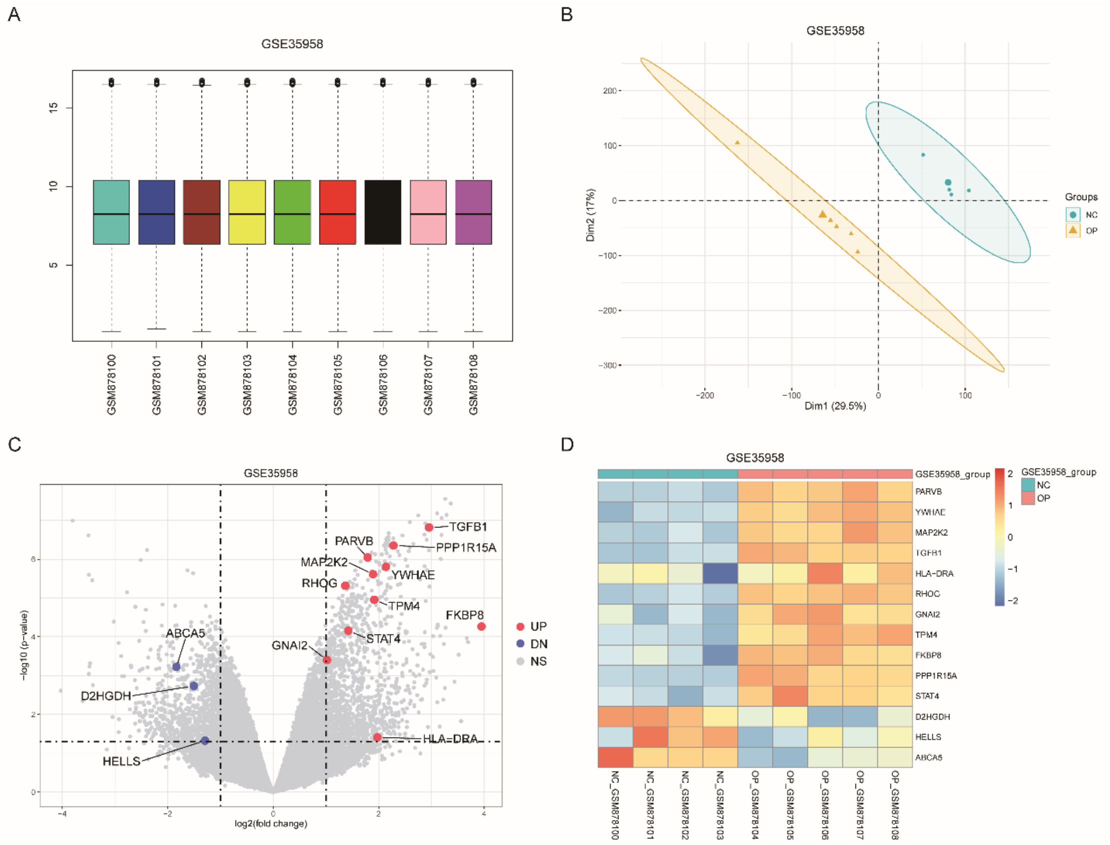

Supplementary Figure *3. Data Pre-processing for the Validation Dataset GSE35958*

GSEA analysis of the validation dataset GSE35958

To further validate the consistency between the GSEA enrichment results of GSE35958 and previous results, the logFC value of the genes in GSE35958 was averaged with the logFC values of the genes in GSE152293 and GSE25958 for GSEA analysis. It was found that the genes were also enriched in pathways such as HALLMARK_HEME_METABOLISM, HALLMARK_MYC_TARGETS_V1, HALLMARK_ALLOGRAFT_REJECTION, HALLMARK_OXIDATIVE_PHOSPHORYLATION, and HALLMARK_P53_PATHWAY (Supplementary Figure 2).

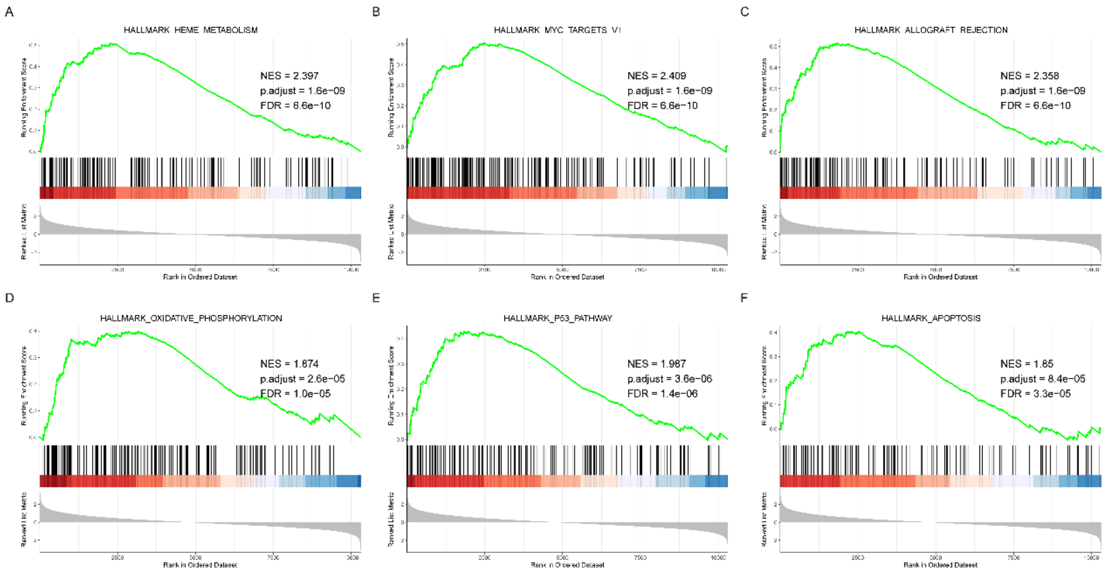

Supplementary Figure 4. GSEA Results for the Validation Dataset GSE35958

ROC analysis

To further explain the relationship of differentially expressed iron metabolism-related genes between the group of patients with osteoporosis and the healthy controls, ROC curves associated with the grouping were created based on the original datasets GSE152293 and GSE35956, and the validation dataset GSE35958, and the AUC were calculated. The results showed that all differentially expressed iron metabolism-related genes demonstrated good performance in the three datasets (Supplementary Figure 3). The AUC of many genes was 1, probably because of overfitting due to the small sample size of this study.

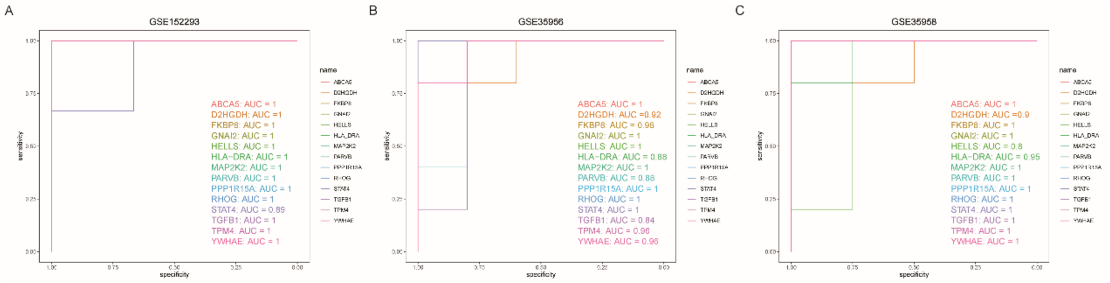

Supplementary Figure 5. ROC Analysis

Figure Legends

Supplementary Figure 1: Point plots of Pearson correlations among samples in GSE152293 (A), GSE35956 (B), and GSE25958 (C) datasets. The color from light blue

to dark blue represents the Pearson coefficient between samples from low to high, and the samples becoming more and more similar. Point size represents the Pearson correlation between samples; the larger the point, the greater the correlation between samples. The red numbers represent Pearson similarity coefficients between samples. OP: Osteoporosis, NC: Normal control.

Supplementary Figure 2. Verification result of GSE56815 data set. Genes expression in A. SE56815 samples. Y-axis represents gene expression values, X-axis represents samples, the green represents healthy controls, and the yellow represents osteoporosis. B. PCA analysis based on GSE56815 gene expression. Green is the healthy control group, and yellow is the osteoporosis group. Differential expression of genes in C. gSE56815.X axis represents log foldchange of gene expression difference, the y axis represents -log(10pvalue), each dot represents a gene, and the gray dot represents insignificant gene expression difference. D. Expression heat map of 21 iron metabolism-related genes in GSE56815. Among them, the color bar represents high or low gene expression, blue represents low gene expression, and red represents high gene expression. Green represents healthy control samples and yellow represents osteoporosis samples. OP: Osteoporosis, NC: Normal control.

Supplementary Figure 3. Data Pre-processing for the Validation Dataset GSE35958. (A) Box diagram of expression distribution between samples in GSE35958. The x-axis represents samples, and the y-axis represents gene expression values. **(B)** PCA reduced-dimension diagram of GSE35958. The x-axis and the y-axis represent the two reduced dimensions. Dots in the diagram represent samples, healthy samples are blue, and osteoporosis samples are orange. **(C)** Volcano plot of differentially expressed iron metabolism-related genes in GSE35958. The x-axis is log2(fold change), and the y-axis is -log10(P-value). Each dot represents a gene; blue means downregulated genes, red means upregulated genes, and gray means genes without significant change in expressions. **(D)** Heat map of differentially expressed iron metabolism-related genes in GSE35958. The color bars at the top represent the two groups of patients. Red means

osteoporosis samples, and green means healthy samples. The blue blocks in the diagram mean low expression, and the red blocks mean high expression.

Supplementary Figure 4. GSEA results of the Validation Dataset GSE35958. (A-F) The same pathways as the top six pathways identified through GSEA in the datasets of GSE152293 and GSE35956. The x-axis is the rank of genes in the differentially expressed gene list, the upper y-axis is the enrichment score, and the lower y-axis is the logFC value. Upregulation is > 0 and downregulation is < 0. All the curves are on the left part, corresponding to logFC > 0, so these pathways are all upregulated ones.

Supplementary Figure 5. ROC Analysis. (A-C) ROC curves and the AUC of differentially expressed iron metabolism-related genes in the datasets of GSE155293, GSE35956, and GSE35968 for the osteoporosis patient group and the healthy control group.

References:

1. Wickham, H.J.W.I.R.C.S., ggplot2. 2011. 3(2): p. 180-185.

2. Barrett, T., et al., NCBI GEO: archive for functional genomics data sets--update. Nucleic Acids Res, 2013. 41(Database issue): p. D991-5.

3. Smyth, G.K., Limma: linear models for microarray data, in Bioinformatics and computational biology solutions using R and Bioconductor. 2005, Springer. p. 397-420.

4. Lê, S., J. Josse, and F.J.J.o.s.s. Husson, FactoMineR: an R package for multivariate analysis. 2008. 25(1): p. 1-18.

5. Kolde, R. and M.R.J.R.p. Kolde, Package ‘pheatmap’. 2015. 1(7): p. 790.

Supplementary Materials 2: Full-length of western blot

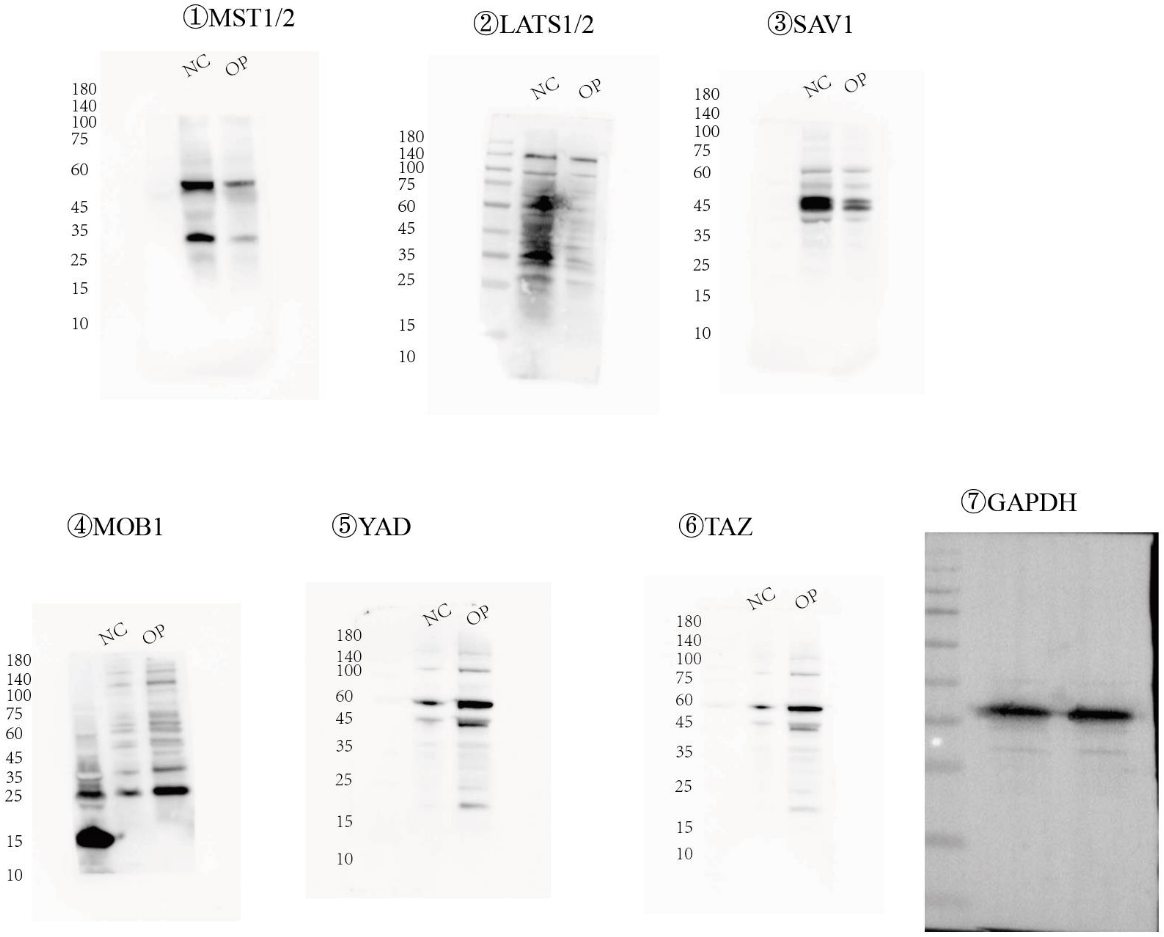

Supplementary Figure 6.Full-length of western blotting showing the expression of related proteins and genes in oxidative stress pathway（Fig 9-C）.

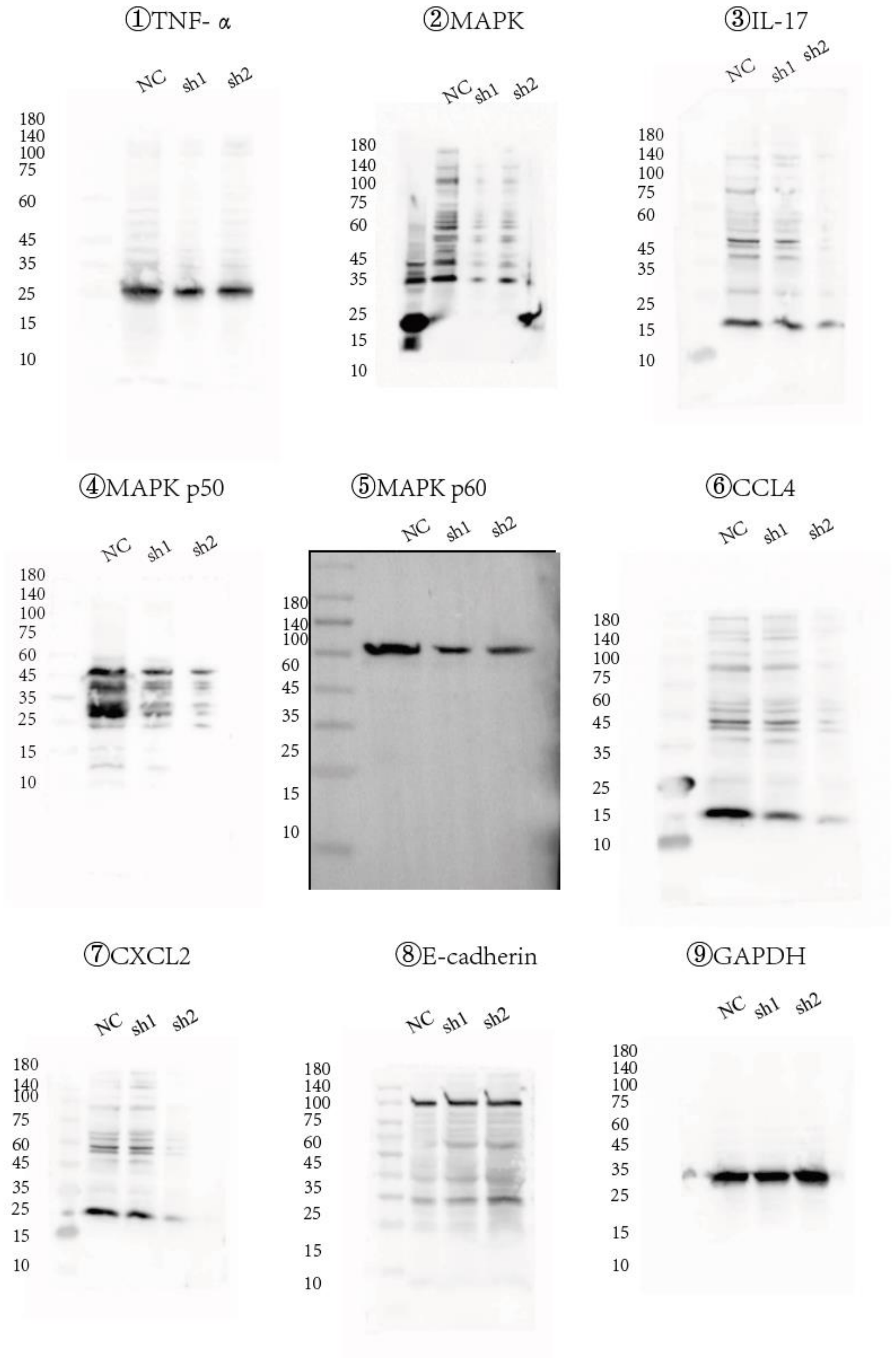

Supplementary Figure 6. Full-length of western blotting showing the expression of downstream pathway related proteins (Fig 9-D).
